# Extracellular vesicles released by non-small cell lung cancer cells drive invasion and permeability in non-tumorigenic lung epithelial cells

**DOI:** 10.1038/s41598-022-04940-6

**Published:** 2022-01-19

**Authors:** Humna Hasan, Ikjot Singh Sohal, Zulaida Soto-Vargas, Anjali M. Byappanahalli, Sean E. Humphrey, Hana Kubo, Sarunya Kitdumrongthum, Sarah Copeland, Feng Tian, Arthit Chairoungdua, Andrea L. Kasinski

**Affiliations:** 1 Department of Biological Sciences, Purdue University, West Lafayette, IN 47907 USA; 2 Purdue Center for Cancer Research, Purdue University, West Lafayette, IN 47907 USA; 3 Toxicology Graduate Program, Faculty of Science, Mahidol University, Bangkok, Thailand; 4 Department of Physiology, Faculty of Science, Mahidol University, Bangkok, Thailand

**Keywords:** Non-small-cell lung cancer, Cancer microenvironment, Multivesicular bodies

## Abstract

Extracellular vesicles (EVs) released from non-small cell lung cancer (NSCLC) cells are known to promote cancer progression. However, it remains unclear how EVs from various NSCLC cells differ in their secretion profile and their ability to promote phenotypic changes in non-tumorigenic cells. Here, we performed a comparative analysis of EV release from non-tumorigenic cells (HBEC/BEAS-2B) and several NSCLC cell lines (A549, H460, H358, SKMES, and Calu6) and evaluated the potential impact of NSCLC EVs, including EV-encapsulated RNA (EV-RNA), in driving invasion and epithelial barrier impairment in HBEC/BEAS-2B cells. Secretion analysis revealed that cancer cells vary in their secretion level, with some cell lines having relatively low secretion rates. Differential uptake of NSCLC EVs was also observed, with uptake of A549 and SKMES EVs being the highest. Phenotypically, EVs derived from Calu6 and H358 cells significantly enhanced invasion, disrupted an epithelial barrier, and increased barrier permeability through downregulation of E-cadherin and ZO-1. EV-RNA was a key contributing factor in mediating these phenotypes. More nuanced analysis suggests a potential correlation between the aggressiveness of NSCLC subtypes and the ability of their respective EVs to induce cancerous phenotypes.

## Introduction

Lung cancer is the leading cause of cancer-related deaths, with non-small cell lung cancer accounting for about 85% of total cases. More people die of lung cancer each year than from colon, breast, and prostate cancers combined^1^. Despite improvements in therapeutic strategies, the 19.4% 5-year survival rate has only marginally improved over the past few decades^2^. Because ~ 80% of the lung cancer cases are diagnosed at a regional or distant metastatic stage, it is important to understand the genetic and microenvironmental factors that contribute to lung cancer progression and metastasis. Indeed, alterations in the RAS, p53, and PI3K pathways^3–5^, mutations in epidermal growth factor receptor (EGFR)^6–8^, as well as microenvironmental factors, such as tumor-associated fibroblasts, macrophages^9–11^, and regulatory T cells^12,13^ have been identified as key mediators of lung cancer progression, recurrence, and metastasis^14^.

In the past decade, extracellular vesicles (EVs) have emerged not only as important mediators of intercellular communication but also as modulators of the microenvironment that are important for maintaining homeostasis and for influencing disease pathogenesis, including cancer metastasis^15^. This heterogeneous group of membrane bound vesicles, classified by their size and pathway of biogenesis, includes apoptotic bodies, microvesicles, and exosomes^16–20^. Tumor derived EVs “educate” healthy cells of the same or distant tissues/organs to enhance cancer progression via epithelial/endothelial barrier modulation, invasion, migration and immune regulation, including formation of pre-metastatic niches in distant healthy sites e.g., lungs and liver^21,22^. Modulation of the recipient cell’s phenotype is driven by biologically active molecules contained in EVs, including lipids, nucleic acids (DNAs, mRNAs, miRNAs, lncRNAs, piRNAs, etc.), and proteins.

In the context of NSCLC, tumor-derived EVs can accelerate angiogenesis and tumor growth in a TGFβ1-dependent pathway^23^ by enhancing VEGF levels via miR-21 transfer^24^, by TIMP-1-induced miR-210 transfer^25^, as well as by miR-23a transfer under hypoxic conditions^26^. NSCLC-derived EVs can also affect lung cancer progression directly by inducing a pro-inflammatory phenotype in mesenchymal stem cells via the NFκB-TLR pathway^27^, which promotes lung cancer growth, or indirectly by affecting the functions of immune cells in the tumor microenvironment^28–30^. NSCLC EVs have also been reported to be intimately involved in driving invasion^31^, migration^32^, and metastasis^15^, and inducing an epithelial-to-mesenchymal transition (EMT)^33–35^. While these studies show how NSCLC-derived EVs promote cancer progression by evaluating distinct aspects of cancer, most of these studies use cancer cells as the targets with few exceptions.

To date, few studies have comprehensively investigated the effects of NSCLC EVs on non-tumorigenic recipient cells such as bronchial epithelial cells. Because cancerous cells are juxtaposed against normal epithelial cells, and EV load is highest in this paracrine-like environment, the effects that cancer cell-derived EVs impose on normal cells of the epithelium is of great interest. Indeed, the airway epithelium helps to maintain homeostasis in the lung, and disruption of the epithelial barrier can lead to the development of inflammation and acute lung injury, and can contribute to lung cancer progression^36–38^. In fact, loss of tight junction proteins responsible for epithelial barrier integrity is correlated with lower survival in lung adenocarcinoma patients^38^. However, it is yet to be known if NSCLC EVs have the ability to impair a mature epithelial barrier. Additionally, it remains unclear how different NSCLC cells vary in their EV-secretion profile, information that is important in these early stages of understanding EV biology..

In this study, we demonstrate how cancer cell-derived EVs from different subtypes of NSCLC – adenocarcinoma (A549), squamous cell carcinoma (SKMES1), bronchioalveolar carcinoma (H358), large cell carcinoma (H460), and aggressively metastatic lung cancer (Calu6) differ in their EV secretion profile in comparison to non-tumorigenic bronchial epithelial cell lines (HBEC and BEAS-2B). We also identify differential uptake of NSCLC EVs by two non-tumorigenic lines, HBEC and BEAS-2B cells. Furthermore, we show for the first time how EVs derived from these NSCLC cells differ in their ability to alter the homeostasis of non-tumorigenic epithelial cells, including promoting invasion of HBEC and BEAS-2B cells and impairing a non-tumorigenic epithelial barrier. The study further reveals the contribution of EV-contained RNA (EV-RNA) in driving some of the observed EV-mediated phenotypes.

## Results

### Isolation and biophysical characterization of extracellular vesicles

**Figure 1:**
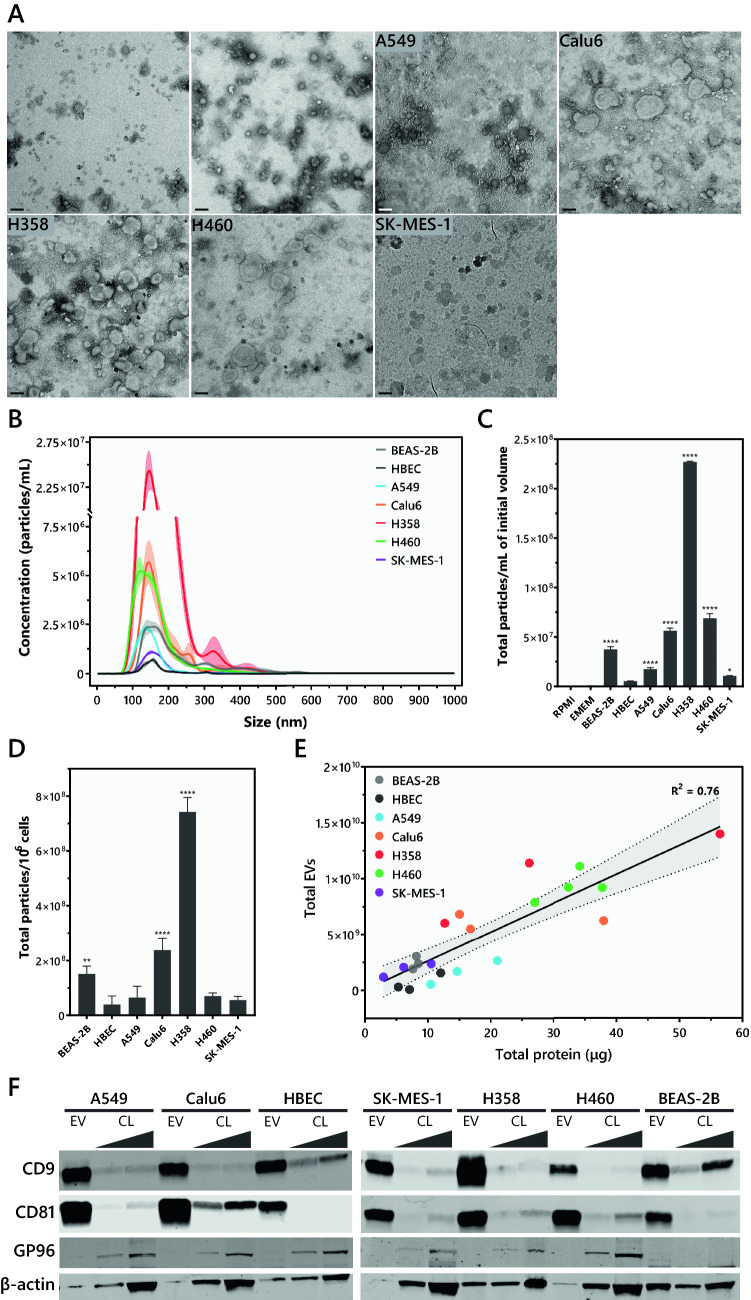
*Biophysical characterization of extracellular vesicles (EVs)*. (**A**) Negative staining and transmission electron microscopy (TEM) of EVs isolated from all cell lines shows vesicle-shaped particles in a similar size range (size bar – 100 nm). (**B**) Size distribution analysis by NTA confirms the size range observed by TEM. (**C**) EV secretion rate evaluation by measuring total particles/mL of initial culture volume. EV-depleted RPMI and EMEM medium were used as negative controls. (**D**) EV secretion rate evaluation by measuring total particles/10^6^ cells. (**E**) Correlation between total EVs and total protein (μg) for all cell lines demonstrates a strong linear correlation with an R^2^ = 0.76 (*p* value < 0.0001). The dotted line represents the 95% confidence interval for the linear correlation. (**F**) Western blotting confirms the presence and enrichment of EV markers (CD9 and CD81) in EV lysate (4 μg total protein) in comparison to cell lysate (CL) loaded at 4 and 25 μg total protein. On the contrary, GP96 – an endoplasmic reticulum protein is enriched in CL but not EV lysate. (Following transfer, membranes were cut, incubated in respective antibodies, and were imaged using the Licor. Cut, uncropped blots, depicting the full region scanned on the Licor can be found in Supplementary Information Fig. S4).

EVs from various non-tumorigenic and NSCLC cell lines were isolated and characterized as per the International Society of Extracellular Vesicles (ISEV) recommendations in MISEV2018^39^. Transmission electron microscopy of isolated EVs confirmed the presence of vesicle-shaped particles in the range of 100–200 nm (Fig. 1A), which was verified by Nanoparticle Tracking Analysis (Fig. 1B). EV secretion rate was also evaluated in all cell lines based on the initial culture volume, as well as initial cell number, and fold change was calculated in comparison to HBEC cells. When normalized to culture volume, all NSCLC cell lines as well as BEAS-2Bs had a significantly higher EV secretion rate in comparison to HBEC cells. H358 had the highest and HBEC had the lowest EV secretion rate. In decreasing order, the EV secretion rate for the various cell lines in comparison to HBEC was H358 (43-fold) > H460 (13-fold) > Calu6 (11-fold) > BEAS-2B (sevenfold) > A549 (threefold) > SKMES-1 (twofold) > HBEC (5.25 × 10^6^ ± 2.8 × 10^5^/mL) (Fig. 1C). When normalized to secretion per million cells, only Calu6, H358, and BEAS-2B had significantly higher EV secretion rates in comparison to HBEC cells (Fig. 1D). A correlation between the number of EVs and EV protein concentration (measured by BCA assay) was also determined to ensure that protein concentration is a good proxy for subsequent EV treatment doses. The correlation between particle number and EV protein concentration was primarily linear with an R^2^ value of 0.76 (Fig. 1E). Finally, CD9 and CD81, which are established EV markers, were found to be significantly enriched in EV lysates (4 μg total protein) in comparison to cell lysates loaded at 25 μg total protein^40^ (Fig. 1F). Conversely, an endoplasmic reticulum marker (GP96) was found to be enriched in total cell lysates but not EV lysates, further confirming the purity of EV isolates^40^. While we extensively characterized EV isolations, following recommendations provided by the ISEV, some non-vesicular contaminants such as protein or DNA could be inadvertently isolated alongside EVs.

### Uptake of NSCLC EVs and their potential to induce invasion in non-tumorigenic epithelial cells

**Figure 2:**
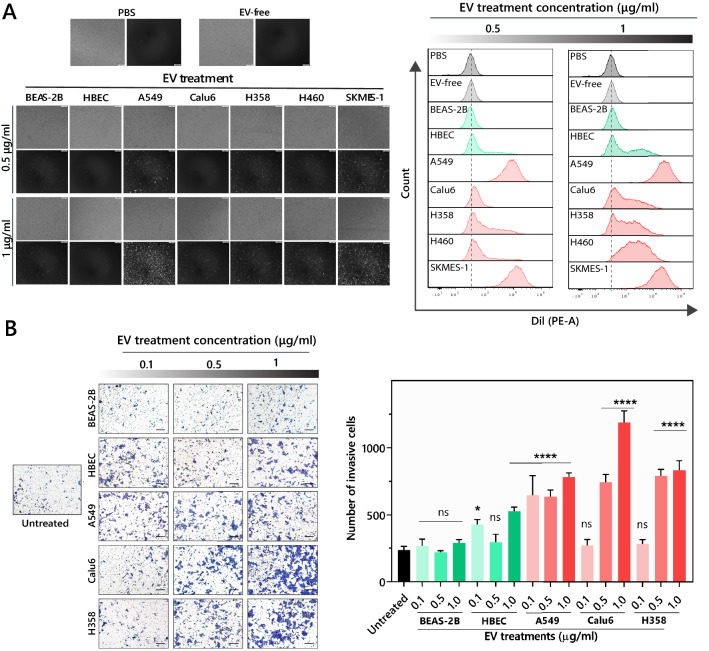
Uptake of NSCLC EVs and their potential to induce invasion in non-tumorigenic epithelial cells. (**A**) Representative fluorescent microscope images were taken with an exposure time of 121.1 ms 48 h after treatment with Dil-stained EVs (left panel). The ‘EV-free’ sample is treated in the same way, but in the absence of EVs to ensure removal of free dye from the samples. Histograms of flow cytometry analysis of BEAS-2B 48 h following treatment with the indicated stained-EVs (right panel). The experiment was reproduced three times with a representative experiment shown. (**B**) Representative images of trans wells showing invasive effect of 0.1, 0.5 and 1 μg/mL doses of tumorigenic (Calu6, A549, and H358) and non-tumorigenic (HBEC and BEAS-2B) cell-derived EVs on BEAS-2B cells (Scale bar, 200 μm). The number of invading cells for all treatment groups were compared to untreated control. p-values were determined using one-way ANOVA followed by Dunnett’s multiple comparison tests (n = 3, *p < 0.05 and ****p < 0.0001).

Extensive evidence suggests that EVs, after being secreted by donor cells, are either internalized by nearby cells or enter into circulation to be taken up by distant tissues^15^. General routes of internalization proposed for EVs include fusion and endocytosis^41,42^. To test EV-mediated responses in recipient cells, we first evaluated uptake of various NSCLC cell line-derived EVs by non-tumorigenic epithelial cells. Uptake of EVs was assessed 48 h following exposure, which signifies the length of duration for which functional responses (including invasion and permeability) were later assayed. Differential uptake of EVs by non-tumorigenic cells (BEAS-2B and HBEC) was observed, which increased in a dose-dependent manner (Fig. 2A). Furthermore, EVs isolated from NSCLC cells lines were generally taken up more readily than EVs from non-tumorigenic epithelial cells, with EVs from A549 and SKMES consistently resulting in the highest uptake by both BEAS-2B (Fig. 2A) and HBEC recipient cells (Supplementary Fig. S1A). While uptake of most EVs was similar between HBEC and BEAS-2B cells, uptake of Calu6 EVs by HBEC cells was higher than uptake by BEAS-2B cells. Thus, not only are various EVs taken up at different rates, the recipient cell line can also dictate uptake kinetics.

Following uptake, we investigated the functional effects of NSCLC EVs in driving invasion, one of the six hallmarks of cancer known to play an important role in tumor development^43,44^. Non-invasive HBECs and BEAS-2Bs were used as recipient cells to quantify if an invasive phenotype could be induced by the EVs. While neither of the non-tumorigenic EV isolates promoted a significant dose-dependent increase in invasion in recipient BEAS-2B, all three NSCLC cell line-derived EVs (A549, Calu6, and H358) significantly enhanced the invasiveness of BEAS-2B cells in a dose-dependent manner (Fig. 2B). EVs derived from H358 and Calu6 cells caused a dramatic increase in invasion in recipient cells, in a dose-dependent manner, with Calu6 EVs, despite their low uptake, driving the most striking invasive response by augmenting the invasive capacity of HBEC and BEAS-2B cells by ~ 2 and fivefold respectively. Even though uptake of Calu6 EVs was slightly higher in HBEC cells than BEAS-2B cells, a similar invasion effect was observed when HBECs were used as recipient cells (Supplementary Fig. S1B), while again, the non-tumorigenic BEAS-2B and HBEC-derived EVs did not alter invasiveness. This evidence indicates that NSCLC EVs can significantly enhance the invasive potential of otherwise non-invasive cells and that the invasive effect induced by Calu6 EVs was more pronounced than any of the other NSCLC EVs.

### EVs from NSCLC cell lines impair epithelial barrier properties

**Figure 3:**
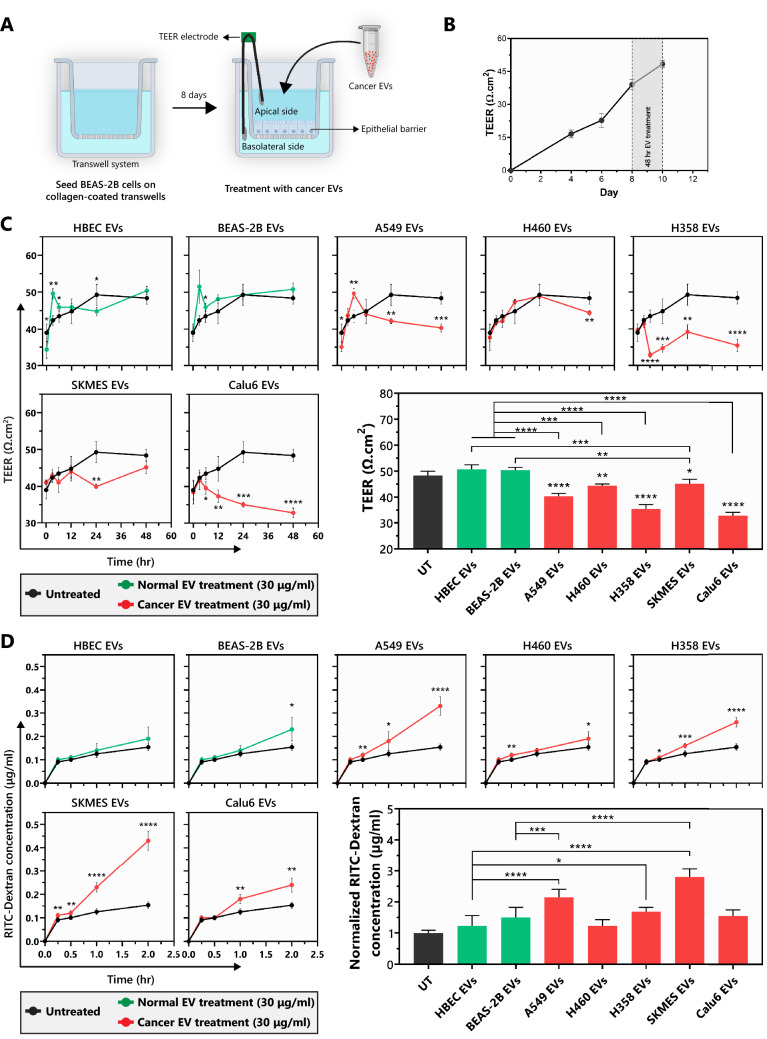
BEAS-2B epithelial barrier impairment mediated by NSCLC EVs. (**A**) Schematic representing the design of epithelial barrier impairment studies. (**B**) Trans-epithelial electrical barrier (TEER) progression of the BEAS-2B epithelial barrier over 10 days. The TEER prior to EV treatment (on day 8) was 39.01 ± 2.4 Ω.cm^2^. (**C**) TEER progression after treatment of an eight-day mature BEAS-2B epithelial barrier with non-tumorigenic or cancer cell derived EVs (n = 3). The bar graph represents TEER at the end of the 48 h after treatment. P-values for TEER progression and bar graph were computed using unpaired t-test with Welch’s correction and ANOVA followed by Dunnett’s multiple comparison tests, respectively. (**D**) BEAS-2B epithelial barrier permeability measured by apical-to-basal translocation kinetics of 70 kDa RITC-Dextran after treatment with normal or cancer EVs (n = 3). The bar graph represents the fold-change in the amount of RITC-Dextran in the basal chamber after 2 h of the 48 h treatment. P-values for dextran translocation kinetics and bar graph were computed using unpaired t-test with Welch’s correction and ANOVA followed by Dunnett’s multiple comparison tests, respectively. The UT is normalized to 1 μg/ml. *p < 0.05, **p < 0.01, ***p < 0.001 and ****p < 0.00001.

Epithelial barrier impairment is involved in various aspects of cancer progression such as initiation and modification of the tumor microenvironment, angiogenesis, extracellular matrix invasion, primary site escape, extravasation, and metastasis^45^. To evaluate the effect that NSCLC EVs impose on an epithelial barrier, BEAS-2B cells were used to form an intact epithelial barrier that has low permeability and a high trans-epithelial electrical resistance (TEER) of ~ 40 Ω.cm^2^. An intact BEAS-2B barrier was left untreated or was treated with EVs isolated from various NSCLC or HBEC/BEAS-2B cells (Fig. 3A–B). The TEER for the untreated, HBEC EV-treated, and BEAS-2B EV-treated barrier continued to increase during the two-day treatment window. For untreated, HBEC, and BEAS-2B EV-treated barriers, the TEER increased by 9 Ω.cm^2^, 16 Ω.cm^2^, and 7 Ω.cm^2^, respectively (Fig. 3C). In contrast, treatment with NSCLC EVs prominently reduced the TEER, albeit to varying extents. Consistent with the invasion data, Calu6 and H358 EVs had the most aggressive effect on the TEER, resulting in ~ 32% and ~ 27% reduction in comparison to the untreated by the end of the 48 h exposure (Fig. 3C). A549, H460, and SKMES-1 EV treatments caused modest decreases in TEER relative to untreated, 16%, 8%, and 7% reduction, respectively. For the A549 EV treatment, the TEER initially increased, comparable to that of HBEC or BEAS-2B EV treatment, but reduced at 48 h. Similarly, SKMES-1 and H460 EVs did not have a significant effect on TEER at early timepoints but caused a slight, yet significant decrease at 24 and 48 h, respectively, following the onset of EV treatment. The data shows that all NSCLC EVs have the potential to impair BEAS-2B epithelial barrier by reducing the TEER, with Calu6 and H358 EVs causing significantly higher barrier disruption than other NSCLC EVs.

After 48 h of EV treatment, BEAS-2B epithelial barriers were further evaluated for barrier permeability following exposure of treated barriers to 70 kDa RITC-tagged dextran beads. Apical-to-basal movement of beads through the barriers was evaluated over time. Intact barriers would be less permeable to the movement of the beads, while any breech of the barrier would facilitate movement to the basal chamber. As expected, dextran bead movement was negligible in HBEC and BEAS-2B EV-treated barriers except when beads were allowed to diffuse through the barrier for 2 h (Fig. 3D). Among NSCLC EVs, the SKMES EV-treated barrier was the most drastically altered. Beads accumulated in the basal chamber ~ threefold more than what was observed for the untreated barrier. A549 EV-treated barriers were also clearly disrupted as reflected by ~ twofold increase in movement of beads across the barrier at the end of the two-hour exposure (Fig. 3D). Significant changes were also observed following Calu6 and H358 treatment, which following 2 h of exposure resulted in 60% and 73% higher dextran translocation in comparison to untreated. When compared to HBEC or BEAS-2B EV-treated barriers: (i) A549 and SKMES EV-treated barriers were highly permeable to the dextran beads 1 h after bead addition and became significantly more permeable 2 h after bead addition. (ii) Calu6 EV-treated barriers were also highly permeable 1 h after bead addition with a modest increase in permeability 2 h after bead addition. (iii) H358 EV-treated barriers were modestly permeable 1 h after bead addition and became highly permeable 2 h after bead addition. iv) H460 EV-treated barriers were modestly permeable 2 h after bead addition (Fig. 3D bar graph and Supplementary Fig. S2). Taken together, the TEER data and the bead permeability assay suggest that nearly all NSCLC EVs cause defective barrier architecture, albeit to different degrees.

### Differential effect of NSCLC subtype EVs on epithelial barrier-related junctional complex proteins E-cadherin and ZO-1

**Figure 4:**
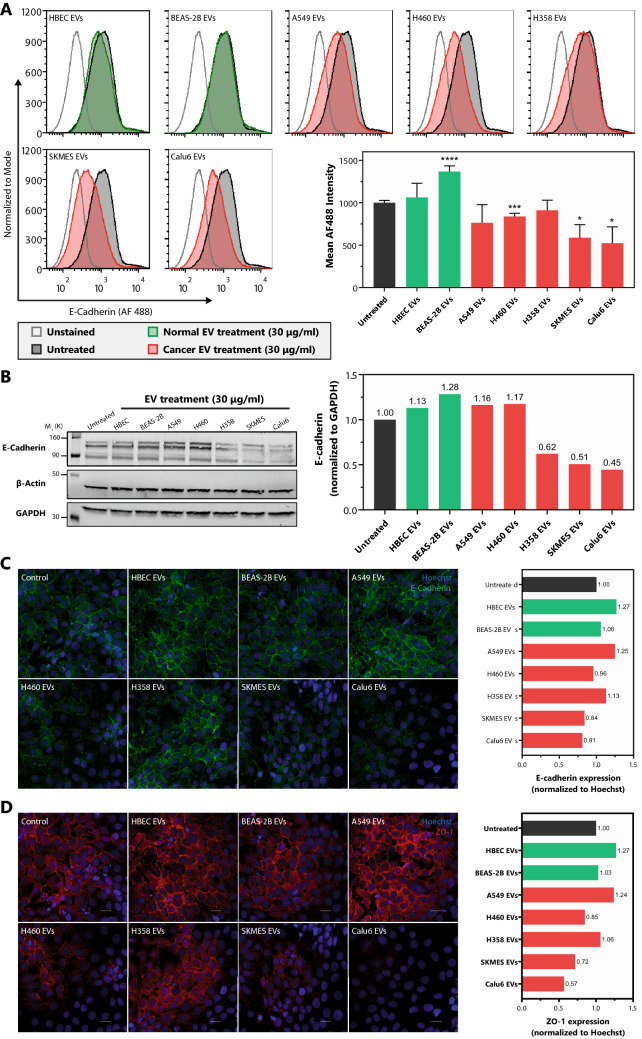
Varied potential of NSCLC EVs to modulate the expression of junctional complex proteins E-cadherin and ZO-1. (**A**) Representative histograms demonstrating the levels of surface E-cadherin on BEAS-2B cells treated with non-tumorigenic or cancer cell derived EVs for 48 h. The bar graph shows quantified data from independent repeats (n = 3). *p* values were computed using unpaired t-test with Welch’s correction. (**B**) Representative western blot showing cellular (surface + intercellular) E-cadherin levels in BEAS-2B cells treated with non-tumorigenic or cancer cell derived EVs for 96 h (Following transfer, membranes were cut, incubated in respective antibodies, and were imaged using the Licor. Cut, uncropped blots, depicting the full region scanned on the Licor can be found in Supplementary Information Fig. S5). Quantification is depicted in the bar graph. Confocal microscopy analysis of (**C**) E-cadherin and (**D**) ZO-1 levels at the intercellular junctional complexes in BEAS-2B cells treated with non-tumorigenic or cancer cell derived EVs for 48 h (size bar – 20 μm). The relative levels from the representative images are normalized to Hoechst stain with the quantification shown on the right for each respective protein. *p < 0.05, **p < 0.01, ***p < 0.001 and ****p < 0.00001.

The integrity of the epithelial barrier and its normal functions are particularly dependent upon the intercellular junctional complexes, such as adherens junctions and tight junctions^45^. Therefore, to further assess the effects of NSCLC EVs on epithelial barrier impairment at the cellular level, the expression of key proteins involved in adherens (E-cadherin) and tight junction (ZO-1) complexes were assessed. Regarding surface E-cadherin expression on BEAS-2B cells, treatment with HBEC EVs did not cause any significant changes. However, treating BEAS-2B cells with their own EVs increased surface E-cadherin expression by 36.8% in comparison to untreated (Fig. 4A). This trend was consistent following evaluation of total cellular (surface + intracellular) E-cadherin levels (Fig. 4B). Among NSCLC EVs, H460, SKMES, and Calu6 EVs caused significant downregulation of surface E-cadherin by 16%, 41.2%, and 47.8%, respectively (Fig. 4A). Similarly, total E-cadherin in cells treated with SKMES and Calu6 EVs was reduced 49% and 55% (Fig. 4B). On the other hand, H460 EVs did not change overall E-cadherin expression significantly; however, based on the significant change in surface E-cadherin, it is possible that H460 EVs may disrupt E-cadherin localization opposed to overall expression. The inverse was observed following treatment with H358 EVs, which did not affect surface E-cadherin expression but generated a significant reduction in total E-cadherin expression in BEAS-2B cells (Fig. 4B). Confocal analysis further confirmed that non-tumorigenic cell-derived EVs (HBEC and BEAS-2B EVs) had no effect on reducing E-cadherin and ZO-1 levels following treatment of BEAS-2B cells. Consistently, SKMES and Calu6 EVs caused significant downregulation of E-cadherin (16% and 19%, respectively) and ZO-1 (28% and 43%, respectively) (Fig. 4C–D). H460 EVs also modestly downregulated E-cadherin and ZO-1, consistent with the flow cytometry data shown in Fig. 4A. The only cancer EVs that did not appear to alter E-cadherin and ZO-1 expression or localization were A549 EVs, suggesting that the increased permeability imposed by A549 EVs (Fig. 3) is likely mediated by another mechanism. Nonetheless, the same EVs that generated the most significant effect on BEAS-2B permeability (H358, SKMES, and Calu6, Fig. 3C), also caused a significant reduction, or change in localization of E-cadherin or ZO-1.

### Assessment of EV-encapsulated RNA as a critical contributor of EV function

EV-mediated modulation of recipient cells is attributed to transfer of diverse bio-active molecules^46^. Specifically, various RNA species enclosed in EVs have been shown to be important determinants of EV-induced responses at local and distant target sites^47–49^. However, the extent to which EV-contained RNA is crucial to EV-induced responses is not known. Therefore, after confirming distinct cancer-related phenotypes induced by EVs, we assessed if any of the functional effects of NSCLC EVs could be attributed to RNAs enclosed in EVs (henceforth termed “EV-RNA”). Before establishing the role of EV-RNA, we first validated that unencapsulated RNA, so-called “EV-free RNA”, does not contribute to the invasion phenotype. To conduct this analysis, it was important to degrade any EV-free RNA that was unintentionally purified during EV preparation, and to reevaluate the effects of the treated EVs on the various phenotypes. We first established a dose of RNase A that results in degradation of unprotected RNAs without effecting intra-exosomal RNAs. To test the effectiveness of RNase A in depleting EV-free RNAs, exogenous miR-34a was added to tubes containing EV preparations to represent EV-free RNA, and miR-451a, an endogenous microRNA previously reported, and confirmed in this study, to be enriched in EVs was used to represent EV-RNA^50^. EV fractions were subjected to various concentrations of RNase A, and the resulting relative abundance of miR-34a was quantified through qRT-PCR. As expected, miR-34a was degraded in a dose-dependent manner (Supplementary Fig. S3A). Based on these results, a final RNase A concentration of 0.5 µg/mL was selected for EV treatments to minimize the degradation of intra-exosomal RNA following membrane lysis (that could inadvertently occur with higher RNase A doses). While this concentration did not completely abrogate extra-exosomal miR-34a, it resulted in a 1,000-fold decrease in miR-34a, which we hypothesized was more than sufficient for reducing EV-free RNA and its effects, if any (Supplementary Fig. S3A). The samples were then treated with an RNase inhibitor at various doses, and EV-RNA was purified followed by quantification of miR-451a levels. The goal was to identify a concentration of RNase inhibitor that was sufficient. Such a dose would result in miR-451a levels similar to untreated EV preparations. In conclusion, incubation with 0.5 µg/mL of RNase A followed by 7.5 U/mL of RNase inhibitor degraded miR-34a (EV-free RNA) but maintained similar levels of miR-451a (EV-RNA) (Supplementary Fig. S3B–C). Thus, these doses were used to generate EVs, free from contaminating unencapsulated RNA.

**Figure 5:**
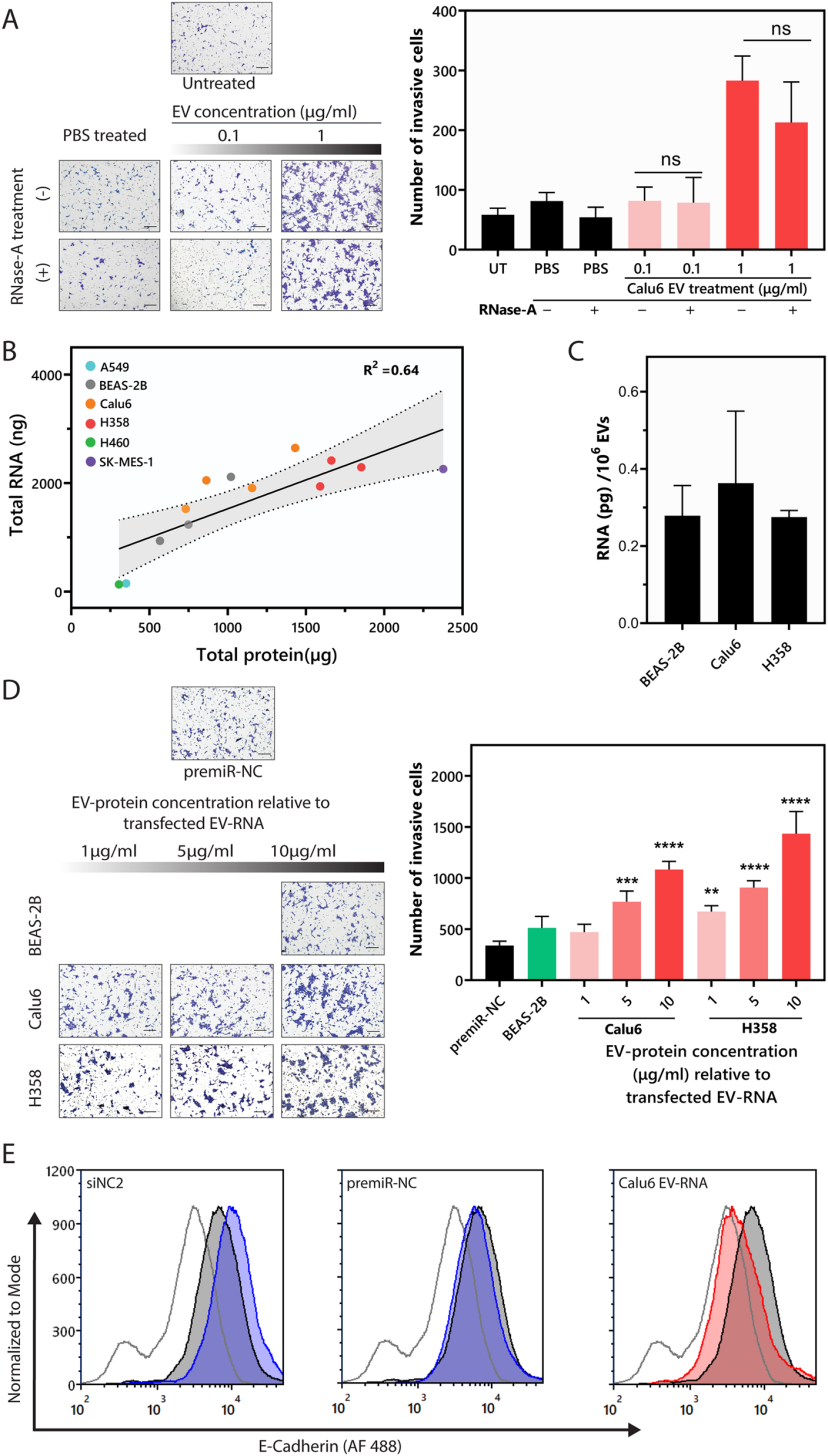
EV-RNA contributes to NSCLC EV-mediated phenotypes. (**A**) Representative images showing the invasive effect of RNase A treated Calu6-EVs on BEAS-2B cells (Scale bar, 200 μm). The number of invasive cells was compared between treatments using unpaired t-test with Welch’s correction. There was no significant difference between BEAS-2Bs incubated with and without RNase A treated Calu6 EVs. Untreated sample is represented as UT (**B**) Correlation between EV protein (µg) and EV-RNA (ng) indicates a strong linear correlation with an R^2^ = 0.64, the dotted line represents the 95% confidence interval for the linear correlation. (**C**) EV-RNA yield per million EVs is not statistically different between tumorigenic (Calu6 and H358) and non-tumorigenic (BEAS-2B) cells. (**D**) Representative images showing the invasive effect of EV-RNA on BEAS-2B cells (Scale bar, 200 μm). EV-RNA concentrations relative to 1, 5, and 10 µg/mL of EV-protein were calculated (see Table S1). BEAS-2B cells were transfected with the indicated *protein-equivalent EV-RNA concentration* and invasion was quantified 48 h following transfection. EV-RNA from NSCLC EVs (Calu6 and H358) significantly increase the number of invasive cells in comparison to premiR-NC. p-values were determined using one-way ANOVA followed by Dunnett’s multiple comparison tests (n = 3, **p < 0.01, ***p < 0.0005 and ****p < 0.0001). (**E**) Representative histograms demonstrate the levels of surface E-cadherin on BEAS-2B cells following transfection with negative control RNA (siNC2 or premiR-NC) or Calu6 EV-RNA relative to 300 µg/mL of EV-protein (n = 2).

To rule out the contribution of EV-free RNA in driving the invasive phenotype in non-tumorigenic BEAS-2B cells, Calu6 EVs treated as above were used. Subjecting BEAS-2B cells to 0.1 and 1 μg/mL of Calu6 EVs, treated with or without RNase A, induced invasion in recipient BEAS-2B cells to similar levels. At 1 μg/mL, the number of invasive cells was 283 ± 41.1 without RNase A treatment and 213 ± 67 with RNase A treatment. These results suggested no significant involvement of EV-free RNA in driving the invasive phenotype (Fig. 5A).

Given that the EV-driven response was not influenced by EV-free RNA, we then assessed if EV-RNAs are crucial to EV-mediated functional effects, and to what extent. To keep the functional contribution of EV-RNA relevant to the EV doses used for earlier phenotypic assays (based on amount of protein), a correlative analysis of EV-RNA and EV-protein was conducted. EV preps from which quantifiable RNA was obtained were used for this analysis. EV-RNA and EV-protein concentration showed a strong linear correlation with an R^2^ value of 0.64 (Fig. 5B). We also assessed the relationship of EV-RNA to particle number and found no significant differences between EV samples. EV-RNA quantification relative to particle number for BEAS-2B, Calu6, and H358 EVs was 0.27 ± 0.08, 0.36 ± 0.18 and 0.27 ± 0.017 pg/10^6^ EVs, respectively (Fig. 5C). Likely, this suggests that while the non-tumorigenic and tumorigenic cell-derived EVs may differ in EV content/cargo, the total amount of EV-RNA is very similar, which was further substantiated by the fact that no significant difference was observed in EV-RNA yield per million EVs for non-tumorigenic (BEAS-2B) and tumorigenic (Calu6 and H358) cells. EV-RNA-induced phenotypic responses were then evaluated for EVs that showed the highest potential for promoting invasion and epithelial barrier disruption i.e., H358 and Calu6 EVs. To determine the degree to which EV-RNA imparts invasion in recipient cells, cells were transfected with RNA concentrations equivalent to 1, 5 and 10 µg/mL of the corresponding protein concentrations (Supplementary Table S1). Transfection with Calu6 and H358 EV-RNA significantly augmented the invasive capacity of BEAS-2B cells (Fig. 5D). Specifically, H358 EV-RNA increased the invasiveness of BEAS-2B cells from 338.6 ± 42.5 (premiR-NC treatment) to 672 ± 56.4 (twofold), 906 ± 67.2 (2.7-fold), and 1434 ± 215.8 (4.2-fold) at 1, 5 and 10 μg/mL *EV-RNA-equivalent protein* dose, respectively. Calu6 EV-RNA increased the number of invasive cells 2 and threefold at 5 and 10 μg/mL *EV-RNA-equivalent protein* dose, respectively, but did not have a significant effect at 1 µg/mL (Fig. 5D). In contrast, BEAS-2B EV-RNA at the highest dose (10 μg/mL) did not alter the invasion of BEAS-2B cells when compared to cells transfected with premiR-NC. Transfecting BEAS-2B cells with Calu6 EV-RNA also significantly downregulated surface E-cadherin levels (Fig. 5E). The data indicate that NSCLC EV-RNA functionally contributes to inducing invasion and barrier modulation in non-tumorigenic recipient cells.

## Discussion

Cancer-derived extracellular vesicles (EVs) play a pivotal role in cancer progression by mediating bi-directional communication between cancer cells and their environment, thereby acting as central mediators of signaling loops that shape the tumor microenvironment^51,52^. In NSCLC, tumor-derived EVs can accelerate angiogenesis and tumor growth^23–26^, affect lung cancer progression directly or indirectly^27–30^, promote invasion^31^, migration^32^, induce epithelial-to-mesenchymal transition (EMT)^33–35^, and collectively, have the potential to promote metastasis^15^. While these studies show how NSCLC-derived EVs promote cancer progression, most of these reports use cancer cells as the target cells. Very few studies have comprehensively investigated the effects of NSCLC EVs on non-tumorigenic bronchial epithelial cells and how different NSCLC cells differ in their EV secretion profile. In this study, we performed a comparative analysis of (i) EV release from non-tumorigenic bronchial epithelial cells and several NSCLC cells as well as (ii) the potential of NSCLC EVs and EV-RNA in driving invasion and epithelial barrier impairment in non-tumorigenic cells. The NSCLC cell lines used in the study were deliberately selected to represent different subtypes of NSCLC—A549 (adenocarcinoma), H460 (large cell carcinoma), H358 (bronchioalveolar carcinoma), SKMES (squamous cell carcinoma) and Calu6 (metastatic NSCLC). The data collected suggest a potential correlation between NSCLC subtype-specific features, such as aggressiveness or inherent metastatic ability, and the respective EV’s ability to induce invasion and barrier permeability in non-tumorigenic bronchial epithelial cells. The study highlights the importance of understanding the impact of cancer EVs on normal cells of the lung epithelium.

The secretion rate of cancer cells is generally reported to be higher than normal/non-tumorigenic cells of the same tissue^53–55^. However, most of the current literature reports EV secretion per mL of media/serum or per flask, which does not take into consideration the number of cells^53–55^. Here, EV secretion rate was quantified by both mL of media as well as by cell number. Consistent with previous reports, when measured per mL of media, the EV secretion rate of all cancer cell lines was higher than that of non-tumorigenic HBEC cells (Fig. 1C). However, when measured per million cells, three out of five cancer cells lines (A549, H460, and SKMES-1) had a similar secretion rate as HBEC cells and only two (Calu6 and H358) had a significantly higher secretion rate. Calu6 and H358 had significantly higher EV secretion rates than BEAS-2B cells as well (Fig. 1D). Clearly, normalizing EV secretion rate per million cells takes into consideration the significantly higher proliferation rate of cancer cells in comparison to non-tumorigenic cells^56^. Furthermore, the two non-tumorigenic cell lines –HBEC and BEAS-2B, differ significantly in their EV secretion, with BEAS-2B EV secretion rate at 3.8-fold/million cells and sevenfold/mL of media higher than that of HBECs. The underlying biology of such drastic differences between EV secretion rate of non-tumorigenic bronchial epithelial cells is not well understood and needs to be further evaluated. While EVs derived from non-tumorigenic and tumorigenic cell lines are reported to differ in their protein content, we found a linear correlation between EV number and total protein among all non-tumorigenic and tumorigenic cell lines indicating that the EVs, regardless of the donor cell line or the secretion rate, do not differ significantly in their concentration of protein (Fig. 1E). The linear correlation also indicates, that irrespective of the parent cell line, EV protein can be used as a proxy for EV number.

Evaluation of the EV uptake by non-tumorigenic bronchial epithelial cells revealed differential uptake, with EVs specifically from A549 and SKMES taken up more readily than the EVs from other cell lines (Fig. 2A). Previous reports have suggested that differences in the rate of EV uptake are influenced by differential expression of surface receptors and/or membrane composition of EVs derived from different donor cells, which contribute to different internalization pathways^15,57^. The specific biomolecules or internalization mechanisms that promote increased uptake of A549 and SKMES EVs by non-tumorigenic bronchial epithelial cells have not been elucidated and need to be further investigated. Interestingly, although Calu6 EVs were taken up less in comparison to other NSCLC EVs, they elicited extreme and significant phenotypic changes in recipient cells.

NSCLC EVs are consistently shown to be implicated in cancer progression by potentiating multiple steps of metastasis including invasion^58–60^. Among the NSCLC EVs tested that augment invasive potential of recipient non-tumorigenic cells, Calu6 EVs drove the most dramatic invasive response in recipient BEAS-2B cells. This could qualify Calu6 cells as the one of the most aggressive NSCLC subtypes and could represent one of the mechanisms used to drive metastasis. Further in vitro and in vivo exploration is needed to delineate pathways that are modulated in the recipient cells by cancer-cell derived EVs. The differential invasive response in recipient cells could be based on differential EV cargo, suggesting selective loading of cancer promoting cargo by Calu6 cells, a very active area of research in the field.

Airway epithelium plays an important role in maintaining homeostasis in the lung, and disruption of the epithelial barrier can lead to the development of inflammation, acute lung injury, chronic obstructive pulmonary disorder (COPD) and lung cancer progression^36–38,61^. NSCLC EVs are known to induce epithelial-to-mesenchymal transition (EMT) in bronchial epithelial cells^33^. However, it is not clear if cancer-derived EVs have the potential to disrupt a mature epithelium that is known to inhibit EMT^62^. Our results showed that NSCLC EVs can disrupt the integrity of an epithelial barrier, and adherens (E-cadherin) and tight junction (ZO-1) proteins involved in barrier integrity are downregulated, indicating that EVs may play an important role in loss of tight junction proteins (such as Claudin-1) that correlates with poor survival of lung adenocarcinoma patients^38^.

We showed that EVs from the high secretion rate cancer cell lines (Calu6 and H358) consistently had a higher propensity to induce various cancerous phenotypes in recipient cells, including invasion (Fig. 2), epithelial barrier disruption (Fig. 3), and E-cadherin downregulation (Fig. 4) than the low secretion rate cancer cell lines (A549 and H460). The cell lines with lower secretion rates still induced various cancerous phenotypes but to a lower extent. Interestingly, the low secreting SKMES-1 EVs generated effects similar to EVs isolated from the higher EV secreting cells in their ability to disrupt an epithelial barrier by reducing the TEER, increasing permeability, and downregulating junctional complex proteins—E-cadherin and ZO-1. This could be attributed to a combination of significantly higher uptake of SKMES-1 EVs by BEAS-2B cells (Fig. 2A) as well as the presence of pro-tumorigenic EV cargo. A549 and H460 did affect TEER and barrier permeability to a varying extent, but had somewhat reduced ability in that they were unable to alter the levels of E-cadherin and ZO-1. It is possible that A549 and H460 may have altered other junctional complex proteins that were not tested in this study. This would suggest that although all the cancer EVs tested in this study have the potential to promote pro-tumorigenic phenotypes, mechanistically they may arrive at that effect in different ways, a hypothesis that still needs to be tested. The strong potential of Calu6, H358, and SKMES, but not A549 and H460, at inducing cancerous phenotypes may also be explained based on their origin, as the former three are derived from metastatic sites and may differ in their EV content^63–65^.

From the limited literature available on epithelial barrier-modulating effects of EVs in lung physiology, studies have shown that EVs derived from HIV-exposed macrophages (containing miR-23a) can disrupt epithelial cell integrity by downregulating ZO-1, and LPS-treated macrophage-derived apoptotic bodies have been shown to promote proliferation of BEAS-2B cells via miR-221/222 transfer^66,67^. Studies pertaining to the intestinal epithelial barrier also report that EVs derived from immune cells such as neutrophils and macrophages, and from endothelial cells play an important role in regulating the epithelial barrier integrity. However, no study has reported the effect of cancer-derived EVs on epithelial barrier integrity and, to the best of our knowledge, this is the first study that evaluated the effect of NSCLC EVs on a mature BEAS-2B epithelial barrier. The question that still remains less understood is whether the NSCLC EV-mediated epithelial barrier impairment in non-tumorigenic epithelial cells is reversible or irreversible.

An intriguing question in current EV biology is which of the bioactive molecules are crucial to EV function. Although extensive literature has reported the importance of EV-associated RNAs (mRNAs, piRNA, Y-RNAs, miRNAs, and other non-coding RNAs) in imparting functional phenotypes^24–26,66,67^, the concentration of RNA present in the EVs and the extent to which EV-RNA contributes to EV-mediated responses in recipient cells is not known. To begin to uncover this, the functional contribution of EV-free RNA and EV-RNA in driving cancerous phenotypes in BEAS-2B cells was evaluated. Calu6 EVs and Calu6 EV-RNA were used for these experiments as Calu6 cells had a significantly higher EV secretion rate than other NSCLC cell lines and Calu6 EVs consistently induced strong pro-tumorigenic effects in non-tumorigenic epithelial cells (Fig. 2B and Supplementary Fig. S1B) as well as effects on the epithelial barrier (Figs. 3 and 4). Our results suggest no significant contribution of EV-free RNA isolated during Calu6 EV preparations in driving EV-mediated invasive response. However, this does not rule out the contribution of EV-free RNA that may be associated with RNA binding proteins, and thus refractory to RNase A degradation at the concentration used that could be eliciting the invasive phenotype in BEAS-2B cells. Based on these results, the contribution of EV-RNA to EV function was evaluated. To keep the functional contribution of EV-RNA relevant to EV-protein, a correlative analysis between EV-protein and EV-RNA was conducted. A strong linear correlation was observed (Fig. 5B) suggesting that the ratio of EV-protein to EV-RNA does not significantly differ between different cell lines (Figs. 1E and 5B–C). To quantitatively elucidate the degree to which EV-RNA imparts EV-mediated responses, BEAS-2B cells were transfected with doses of EV-RNA that correspond to EV-protein concentrations (Supplementary Table S1). While the transfected RNA resulted in a similar invasive phenotype as treatment with intact EVs, the EV-RNA concentration that was needed to observe a similar effect was ~ tenfold higher than intact EVs (Figs. 2B and 5D). Similarly, Calu6 EV-RNA caused a downregulation of surface E-cadherin levels, albeit the effect was only observed using a similar tenfold higher RNA concentration than the dose of Calu6 EVs that caused the same phenotype. While the difference in concentration between EV-RNA and intact EVs appears to be high, we cannot rule out the possibility that the full effect of the EV-RNA is not observed due to low transfection efficiencies. Our unpublished studies indicate that these non-tumorigenic cell lines are transfected at < 80%; therefore, it is unlikely that the full concentration of EV-RNA supplied to the cells was delivered. Nonetheless, these results allow us to conclude that EV-RNA certainly contributes to, but is not the only driver, in inducing EV-mediated effects^24–26,66,67^. Built on these findings, it is pivotal to identify cancer promoting RNAs and non-RNA cargo contained in NSCLC EVs which may reveal the pathways that are altered in the recipient cells. It is also critical to gain a more comprehensive understanding of how various RNAs are packaged into EVs. Indeed, recent reports have identified mediators involved in packaging of miRNA into EVs, including hnRNPA2B1, SYNCRIP, MVP, and GW182^68–71^. As we move closer to realizing the clinical benefit of miRNA therapeutic approaches^72,73^, a thorough understanding of these, and other mechanisms that cells use to redistribute miRNAs into the tumor microenvironment is critical.

**Figure 6:**
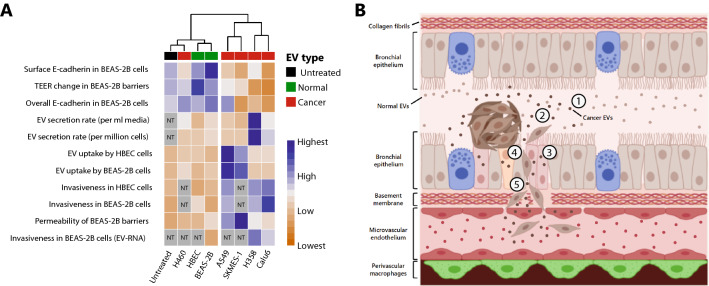
Overall summary of the study and key findings*.* (**A**) Data compiled from all the experiments in the study is summarized in the form of a heatmap. Hierarchical clustering indicates the differences in EV secretion rate, EV uptake, and the potential of various NSCLC EVs and EV-RNA to induce pro-tumorigenic transformations in HBEC/BEAS-2B cells (distance method = euclidean, clustering method = ward.D2). Grey boxes indicate samples that were not tested/evaluated (NT). TEER change in BEAS-2B barriers ranges from positive change (blue) to negative change (orange). **(B)** Key findings from the study include the following: (1) Biophysical and molecular analysis of EVs determined that the EV secretion rate for some NSCLC cell lines is lower than secretion rates from non-tumorigenic bronchial epithelial cells. It was also determined that EVs from non-tumorigenic cells and NSCLC cells have similar total RNA and total protein content per EV. (2) EVs from the various NSCLC cell types differ in their uptake by non-tumorigenic cells. (3) NSCLC EVs as well as EV-RNA can disrupt a BEAS-2B epithelial barrier and downregulate intercellular junctional complex proteins; (4) NSCLC EVs and EV-RNA can impart an invasive phenotype in non-tumorigenic HBEC and BEAS-2B cells.

In conclusion, our study provides a comprehensive summary of the nature of EV secretion in non-tumorigenic and NSCLC cells as well as the impact of NSCLC EVs on the transformation of non-tumorigenic bronchial epithelial cells, which provides a renewed perspective to the understanding of EV biology in cancer. Our comparative data analysis indicates that, when normalized to cell number, several NSCLC cell types have EV secretion rates lower than that of non-tumorigenic cells. Interestingly, the ones which do have higher EV secretion rates (Calu6 and H358), consistently demonstrate greater ability in promoting a tumorigenic response in non-tumorigenic epithelial cells. SKMES-1 was an exception as it had a low EV secretion rate but strongly induced a cancerous phenotype, likely due to the combination of significantly high uptake as well as oncogenic EV content. Furthermore, EVs, regardless of being isolated from non-tumorigenic cells or from NSCLC cells, demonstrate a very similar biological composition in terms of total RNA and total protein content. Additionally, EV-RNA stands out as an important constituent of EVs. The study and its overall findings are summarized in Fig. 6.

While epithelial cells were used as recipient cells in this study, it remains to be seen how NSCLC EVs affect other cells associated with the normal lung epithelium such as mucus-secreting, basal, immune, neuroendocrine and other rare cell types. This study lays the groundwork needed to begin to perform additional analysis to address important questions related to basic EV biology and the role of EVs in NSCLC progression, such as the underlying (i) mechanism(s) involved in differential uptake of NSCLC EVs by non-tumorigenic epithelial cells; (ii) mechanisms by which different NSCLC EVs promote pro-tumorigenic phenotype in non-tumorigenic epithelial cells (e.g. selective packaging of EV cargo, types of EV cargo, mutations in cancer cells, etc.); (iii) mechanism(s) of differential EV secretion between non-tumorigenic epithelial cells; and (iv) long-term effects of NSCLC EV-mediated transformation. It is also important to understand how cells with differential EV secretion rates differ in terms of EV content and EV biogenesis. The findings also highlight the need to identify specific EV-RNAs and other cargo involved in driving normal epithelial cell transformation.

## Methods

### Equipment and settings

#### Nanoparticle tracking analysis

Nanoparticle tracking analysis was conducted using NanoSight LM10 (Malvern Panalytical Inc., Westborough, MA) equipped with a 405 nm laser. For each EV prep, three 60 s videos were recorded at camera level 8. Temperature was monitored during recording. To determine the size distribution and concentration of measured particles with corresponding standard error, recorded videos were analyzed using NTA Software version 3.3 with detection threshold set to 3. A medium viscosity of 0.929 cP was assumed.

#### Transmission electron microscopy

For TEM imaging specifications, please refer to the Transmission Electron Microscopy section.

#### Western blot imaging

For acquiring western blot images using Li-Cor Odyssey CLX instrument, image studio software was used for qualitative and quantitative measurements of signals. Images were acquired with blots facing down on scan bed and a set focus offset of 0–0.5 mm. Acquisition time varied based upon the size of the blot. Normalization was based upon loaded protein stain (β-actin or GAPDH) captured in appropriate channel (700 nm/800 nm) for fluorophore labelled secondary antibody while protein ladder (Chameleon 700) was captured in 700 nm channel. All blot images were acquired in high quality setting in the software. For all western blots, fllowing transfer, membranes were cut, incubated in respective antibodies, and were imaged using the Licor. Cut, uncropped blots, depicting the full region scanned on the Licor can be found in Supplementary Information).

#### Confocal microscopy

For confocal microscopy, images were acquired using a Nikon A1R-MP multiphoton confocal microscope with a 40X oil immersion objective (Nikon Inc., Melville, NY, U.S.A), using standard excitation and emission fluorescence channels for AlexaFluor 488, AlexaFluor 647 and Hoechst and ND filter set to 4. The images were acquired and analyzed using the Nikon NIS Elements software in the “.nd2” format. The acquisition settings were 2 K × 2 K resolution (pixels) with a scanning frame rate of 1/16 s. The display lookup table (LUT) settings were set to 200–1000 for Hoechst, 300–2000 for AlexaFluor 488 and 1000–3000 for AlexaFluor 647 for all images exporting the files and mean channel intensities.

#### Flow cytometry

Flow cytometry assays were performed using Fortessa Cell Analyzer using the following parameters. For uptake assay experiments, the voltages were set at 253, 200, 466 for FSC, SSC and PE channels respectively. For E-cadherin expression analysis, samples were analyzed with the voltage for FSC, SSC and FITC channel set to 280, 200 and 360, respectively. A total of 1 × 10^4^ cells were analyzed for each sample. Flow cytometry data was analyzed using FCS Express 7 Software.

### Antibodies

The following antibodies were used in their respective experiments; anti-CD9 rabbit monoclonal antibody (Cell Signaling, cat no. 13174), anti-CD81 mouse monoclonal antibody (Thermo Fisher Scientific, cat no. 10630D), anti-Grp94/GP96 rabbit polyclonal antibody (Cell Signaling, cat no. 2104), anti-β-actin mouse monoclonal antibody (Cell Signaling, cat no. 3700S), E-cadherin rabbit IgG polyclonal antibody (Proteintech Inc., cat no. 20874-1-AP), and ZO-1 rabbit IgG polyclonal antibody (ABclonal Inc., cat no. A0659) as primary antibodies. AlexaFluor 488-conjugated goat anti-rabbit IgG (H + L) secondary antibody (Fisher Scientific Inc., cat. no. A11034), Hoechst 33342 (Fisher Scientific Inc., cat no. H3570), Licor IRDye 680RD Goat anti-Mouse IgG (cat no. 926-68070) or Licor IRDye 800CW Goat anti-rabbit IgG (cat no. 926-32211) were used as secondary antibodies.

### Cell culture

Non-small cell lung cancer cell lines (A549, Calu6, H358, H460 and SKMES-1) and non-tumorigenic bronchial epithelial cell line (BEAS-2B) were acquired from American Type Culture Collection (ATCC). The human bronchial epithelial cell line (HBEC) was a gift from John Minna’s lab. A549, Calu6, H358, and H460 were maintained in HyClone™ RPMI 1640 Media (Fisher Scientific, cat no. 16777-145), and SKMES-1 in Corning™ Eagle's Minimum Essential Medium (MEM) (cat no. MT10009CV). HBEC cells were maintained in Gibco™ Keratinocyte-SFM (Thermofisher Scientific, cat no. 17005042). NSCLC cell lines were supplemented with 10% Fetal Bovine Serum. FBS used for EV isolation (EV-depleted FBS) was prepared in 1:1 dilution with the respective media, centrifuged at 110,000xg for 18 h, and supernatant was processed through a Nalgene Thermo Rapid Flow 0.2 µm filter units (Fisher Scientific, cat.no 09-741-02). All cell lines were supplemented with 1% Cytiva HyClone™ Penicillin Streptomycin (Fisher Scientific, cat no. SV300-10) and maintained at 37 °C in 5% CO_2_ humidified incubator.

### Extracellular vesicle isolation

Cells were cultured in recommended media. At 80% confluency, the media was replaced with EV-depleted media. After 48 h, media was collected and centrifuged sequentially at 2,000×*g* for 10 min (to remove cells), 10,000×*g* for 40 min (to remove cell debris and large vesicles) and at 110,000×*g* for 90 min to pellet EVs. The resulting EV pellet was washed in 1X PBS and centrifuged again at 110,000×*g* for 90 min. The final pellet was resuspended in 1X PBS and stored at − 80 °C until further use. All centrifugation steps were performed at 4 °C^40^.

### Nanoparticle tracking analysis

EV isolations were analyzed as previously reported^74^. Briefly, Nanoparticle tracking analysis was conducted using NanoSight LM10 (Malvern Panalytical Inc., Westborough, MA) equipped with a 405 nm laser. For each EV prep, three 60 s videos were recorded at camera level 8. Temperature was monitored during recording. To determine the size distribution and concentration of measured particles with corresponding standard error, recorded videos were analyzed using NTA Software version 3.3 with detection threshold set to 3. A medium viscosity of 0.929 cP was assumed. For optimal measurements, samples were diluted with 1X PBS until particles/frame were within the recommended range of the NTA Software (30–100 particles/frame). All NTA-based size distributions correspond to the hydrodynamic diameters of the particles in suspension.

### Transmission electron microscopy

Samples were deposited onto glow-discharged carbon-coated 200-mesh copper grids (Electron Microscopy Sciences, CF200-Cu) in the presence of 2% PTA (phosphotungstic acid) at a 1:1 ratio. After 30 s, excessive moisture was wicked off using filter paper. The grid was air-dried and imaged using Tecnai T12 transmission electron microscope at 100 keV using Gatan US1000 2 K × 2 K CCD camera.

### Western blotting

Western blotting was performed as described previously^72^. Briefly, cells were lysed for 30 min in RIPA buffer containing 1X protease inhibitor (Fisher Scientific, cat no. PIA32955) and centrifuged for 10 min at 14,800 rpm at 4 °C. Protein concentration for cell lysates and EVs were measured using the BCA protein assay kit (Life Technologies, cat no. 23225). EV lysates (4 μg/lane) and cell lysates (4 μg/lane and 25 μg/lane) were diluted in 1X Laemmli sample buffer (Fischer Scientific, cat no. BP-110NR-25ML) and were resolved on a 12% Polyacrilamide Gel (Bio-rad, cat no. 567-1043). The gel was transferred to a PVDF membrane (Fisher Scientific, cat no. IPFL00010) and subsequently blocked with Odyssey Blocking buffer (Licor, cat no. 927-40003) for 1 h at room temperature. Blocked membranes were incubated overnight at 4 °C with the following primary antibodies; anti-CD9 (1:1000), anti-CD81 (1:250, non-reducing conditions), anti-Grp94/GP96 (1:1000) and anti-β-actin (1:1000). Membranes were washed four times with 1X PBST and incubated for 1 h at room temperature with the following secondary antibodies: Licor IRDye 680RD Goat anti-Mouse IgG (1:10,000) or Licor IRDye 800CW Goat anti-rabbit IgG (1:10,000). After incubation with secondary antibodies, membranes were washed four times with 1X PBST and three times with 1X PBS, and signal was visualized with Li-Cor Odyssey CLX.

### Uptake assays

EVs were labeled with 2 μL of 50 μM Dil dissolved in 100% ethanol (Fisher Scientific, cat no. 04-355-226). Unincorporated dye was removed by processing the samples through Invitrogen™ Exosome Spin Columns (Fisher Scientific, cat no. 4484449). Non-tumorigenic epithelial cells were seeded at 1 × 10^5^ per well in a 24-well plate. After 24 h, cells were co-cultured with dil-stained EVs. After 48 h of EV treatment, cells were washed with PBS and images were taken at 10X (121.1 ms exposure) using a fluorescence microscope (Olympus IX 73). Cells were trypsinized, centrifuged at 1000 rpm at 4 °C for 5 min and resuspended in 300 μL of PBS supplemented with 1% FBS. Flow Cytometry using the BD Fortessa Cell Analyzer was performed for 1 × 10^4^ cells. Data was gated based on forward scattering (FSC) and size scattering (SSC), and the PE-A mean fluorescence intensity was calculated for each sample (refer to Equipment and Settings for more details about the instrument settings).

### Transwell invasion assays

Human Bronchial epithelial cells (HBECs and BEAS-2Bs) were seeded in a 6-well plate at a density of 3 × 10^5^ cells/well and allowed to adhere. HBECs and BEAS-2Bs were incubated with 0.1 µg/mL, 0.5 µg/mL or 1 µg/mL of tumorigenic and non-tumorigenic cell-derived EVs for 24 h. Untreated cells were used as control. Treated HBECs and BEAS-2Bs were trypsinized and seeded on top of hydrated invasion chambers (Corning BioCoat Matrigel Invasion Chamber 8.0 Micron Matrigel matrix, cat no. 354480) in triplicates at a density of 1 × 10^5^ cells per well in 400 μl of supplement-free KSFM and BEBM media, respectively. The cells were allowed to invade through the Matrigel-coated chambers under the influence of a chemoattractant for 24 h. The chemoattractant varied based on the cell type being used. For HBECs, Gibco™ Keratinocyte-SFM (Fisher Scientific, cat no. 17-005-042) supplemented with 20X the recommended culturing concentration of EGF (100 ng/mL) was used. For BEAS-2B cells, complete BEBM Basal Medium (Fisher Scientific, cat no. NC9202780) was used as a chemoattractant. Following invasion, the non-invading cells on the upper surface of the membrane were removed with a cotton swab. Invasive cells were stained using the Differential Quik III Stain Kit (Polysciences, cat no. 24606-250), imaged, and counted using Image J software.

To quantify the invasive phenotype induced by extracellular vesicle-encapsulated RNA (EV-RNA), 3 × 10^5^ cells were seeded in a 6-well format. Twenty-four hours later, cells were transfected with total EV-RNA at concentrations corresponding to 1, 5, or 10 µg/mL of EV-protein. For transfection, Pre-miR™ miRNA Precursor Molecules—Negative Control (premiR-NC) (Life Technologies, cat no. AM17111) was used as a control. The cells were transfected consistently with a total of 42 ng of RNA using Lipofectamine 2000 (Fisher Scientific, cat no. 11-668-019) (see Table S1). Importantly, the final RNA concentration for each transfection was supplemented with premiR-NC to maintain an equal concentration of RNA in each transfection reaction. Twenty-four hours post transfection, cells were reseeded into the upper compartment of trans wells in triplicates and allowed to invade through Matrigel coated chambers as described above.

### Barrier permeability experiments

The 0.4 μm pore-size apical chambers of a 12-well transwell were coated with 8 μg/cm^2^ type I collagen (Fisher Scientific, cat no. CB-40231). BEAS-2B cells were seeded at a density of 2.5 × 10^4^ cells/well in the collagen-coated apical chamber and cell-free medium was added in the basolateral chamber. The medium was changed and TEER measurements were recorded every 2 days for a total of 8 days. Maturation of the BEAS-2B monocultures into an intact epithelium was confirmed by stable TEER (trans-epithelial electrical resistance) and tight junction expression (TJ) of E-cadherin. On day 8, BEAS-2B monolayers were treated with 30 μg/mL of the respective EV samples and changes in TEER, barrier permeability, and TJ expression of E-cadherin were evaluated after 48 h. For TEER measurements, fresh culture media was added, and the cultures were allowed to equilibrate to room temperature. A sterilized and dried chopstick electrode for an EVOM2™ voltohmmeter (World Precision Instruments Inc.) was immersed in a 15 mL tube containing complete media to allow the instrument to stabilize. TEER was measured by immersing one electrode prong in the apical media, and the other prong in the basolateral media of the transwell (see Fig. 3A). TEER values for untreated and experimental transwells were corrected by subtracting TEER values for blank transwell (without cells). TEER values were measured in Ohms (Ω) and normalized to the surface area of the well (Ω.cm^2^). All measurements were done in triplicate. At the conclusion of the TEER experiment, resulting barrier permeability was evaluated by adding 100 μg/mL of 70 kDa RITC-Dextran to the apical chamber and collecting basolateral media over time. A Synergy™ Neo microplate reader (Biotek Instruments, Inc.) was used to evaluate the fluorescence intensity of the collected basolateral media.

### RNA isolation from EVs

Total RNA from EVs was extracted using miRVana RNA isolation kit according to the manufacturer's instructions (Thermofisher-AM1560). RNA was eluted from column in 30μL of nuclease free water and stored at − 80 °C followed by a 15 min DNase I treatment at room temperature (Qiagen 79,254). Total EV derived RNA was quantified by NanoDrop (Thermo Fisher Scientific, Inc.), Qubit Fluorometer (Thermo Fisher Scientific, Inc.), and Agilent Bioanalyzer 2100 (Agilent Technologies, Inc.).

### RNase treatment of EVs

The concentration of RNase A used for RNase treatment of EVs was determined following the addition of a known concentration (16 nM) of unprotected miR-34a into tubes containing EVs and incubating with varying concentrations of RNase A. qRT-PCR analysis following RNase A treatment of unprotected miR-34a and EV protected RNA (i.e., miR-451a) identified the lowest concentration of RNase A that resulted in depletion of unprotected miR-34a while maintaining the concentration of EV-protected miR-451a, in this case 0.5 µg/mL (Fig. S3).

For selected experiments, EVs were treated with 0.5 µg/mL of RNase A to degrade RNA species outside EVs. Aliquots of isolated EVs were thawed on ice. Thawed samples were incubated with 0.5 µg/mL RNase A for 10 min at 37 °C. Following RNase treatment, the samples were incubated with 7.5 U/mL RNase inhibitor (SUPERase•In™ RNase Inhibitor cat no. AM2696) for 30 min at room temperature. EV-RNA was isolated as above and processed for qRT-PCR.

### Confocal microscopy

BEAS-2B cells were grown on 24-well transwell inserts and treated with EVs at 30 μg/mL after 6 days. Inserts were then transferred to a fresh 24-well plate for staining. All washes and staining steps were performed at room temperature with gentle rocking. Cells were washed 5 × 10 min with 200 µL PBS, incubated for 15 min with 200 µL 4% paraformaldehyde (Fisher Scientific Inc., cat. no. AAJ61899AK) to fix, washed 2 × 10 min with PBS, incubated with 100 µL of 0.5% Saponin (Fisher Scientific Inc., cat no. 55-825-5) in PBS for 10 min to permeabilize, washed 4 × 5 min with PBS, and incubated in blocking buffer (1% BSA + 0.1% Saponin in PBS) for 30 min. Cells were then incubated with 100 µL of anti-E-cadherin or anti-ZO-1 rabbit IgG polyclonal antibody at 5.5 μg/mL in blocking buffer for 3 h, washed 5 × 10 min with 200 µL blocking buffer, incubated with AlexaFluor 488-conjugated goat anti-rabbit IgG (H + L) secondary antibody (and 4 µg/mL and Hoechst 33,342 at 10 µg/mL in blocking buffer for 2 h and washed 5 × 10 min with PBS. Membranes were then cut from the transwell inserts using scalpel and forceps, mounted on glass slides in Prolong™ Glass Antifade reagent (Fisher Scientific Inc., cat no. P36982), and covered with a glass coverslip. Confocal fluorescence images were acquired using a Nikon A1R-MP multiphoton confocal microscope with a 40X objective (Nikon Inc., Melville, NY, U.S.A), using standard excitation and emission fluorescence channels for AlexaFluor 488 and Hoechst. The images were acquired and analyzed using the Nikon NIS Elements software in the .nd2 format. The acquisition settings were 2 K x 2 K resolution (pixels) with a scanning frame rate of 1/16 s. All images were set to the same display lookup table (LUT) settings before exporting the files and mean channel intensities.

### Flow cytometry

Flow cytometry was conducted as previously described^75^. BEAS-2B cells were seeded at a density of 5 × 10^4^ cells/mL in 24-well plates and were treated with 30 μg/mL of EVs after 6 days. After 48 h of EV treatment, the cells were trypsinized, centrifuged at 10,000 rpm for 5 min at 4 °C, and incubated in 100 μL of anti-E-cadherin rabbit IgG polyclonal antibody at 5.5 μg/mL (1:100 dilution) in 1X PBS for 1.5 h on ice. After primary antibody incubation, cells were washed with 900 μL of 1X PBS and the centrifuged pellet was incubated in 100 μL of AlexaFluor 488-conjugated goat anti-rabbit IgG (H + L) secondary antibody at a final concentration of 4 µg/mL (1:500 dilution) in 1X PBS for 30 min on ice. After secondary antibody incubation, the cells were washed, centrifuged, and the pellet was resuspended in 500 μL of 1X PBS. Untreated BEAS-2Bs were used as positive control and cells with no primary antibody incubation were used as a negative control. The cell suspensions were analyzed using BD Fortessa Cell Analyzer with the voltage for FSC, SSC and FITC channel set to 280, 200 and 360, respectively.

### Statistical analysis

All experiments were performed in triplicates except where specified. GraphPad Prism software (version 9.0.1) was used for statistical analyses. Unpaired t‐tests with Welch’s correction were used to compare the averages of two groups. Analysis of Variance (ANOVA) was performed followed by Dunnett’s multiple comparison tests to analyze data for more than two groups. Results are reported as Mean ± standard deviation. *p* < 0.05 was regarded as statistically significant. Heatmap and hierarchical clustering analysis of the overall data was conducted using R (v 4.0.4) and R Studio (v 1.4.1106) with the “pheatmap” package.

## Supplementary Information

Supplementary Information.

## Data Availability

All data generated or analyzed during this study are included in this published article (and its Supplementary Information files). The raw files generated during the current study are available from the corresponding author on reasonable request.
