# Author Correction: Extracellular vesicles released by non-small cell lung cancer cells drive invasion and permeability in non-tumorigenic lung epithelial cells

**DOI:** 10.1038/s41598-022-06790-8

**Published:** 2022-02-14

**Authors:** Humna Hasan, Ikjot Singh Sohal, Zulaida Soto-Vargas, Anjali M. Byappanahalli, Sean E. Humphrey, Hana Kubo, Sarunya Kitdumrongthum, Sarah Copeland, Feng Tian, Arthit Chairoungdua, Andrea L. Kasinski

**Affiliations:** 1 Department of Biological Sciences, Purdue University, West Lafayette, IN 47907 USA; 2 Purdue Center for Cancer Research, Purdue University, West Lafayette, IN 47907 USA; 3 Toxicology Graduate Program, Faculty of Science, Mahidol University, Bangkok, Thailand; 4 Department of Physiology, Faculty of Science, Mahidol University, Bangkok, Thailand

Correction to: *Scientific Reports* 10.1038/s41598-022-04940-6, published online 19 January 2022

The original version of this Article contained errors.

The spelling of the author Anjali M. Byappanahalli which was incorrectly given as Anjali M. Byappanhalli.

In addition, in Figure 5A, the image for RNasa-A, 0.1 EV concentration was a duplication of RNasa-A, PBS treated. The original Figure 5 and accompanying legend appear below.

**Figure 5:**
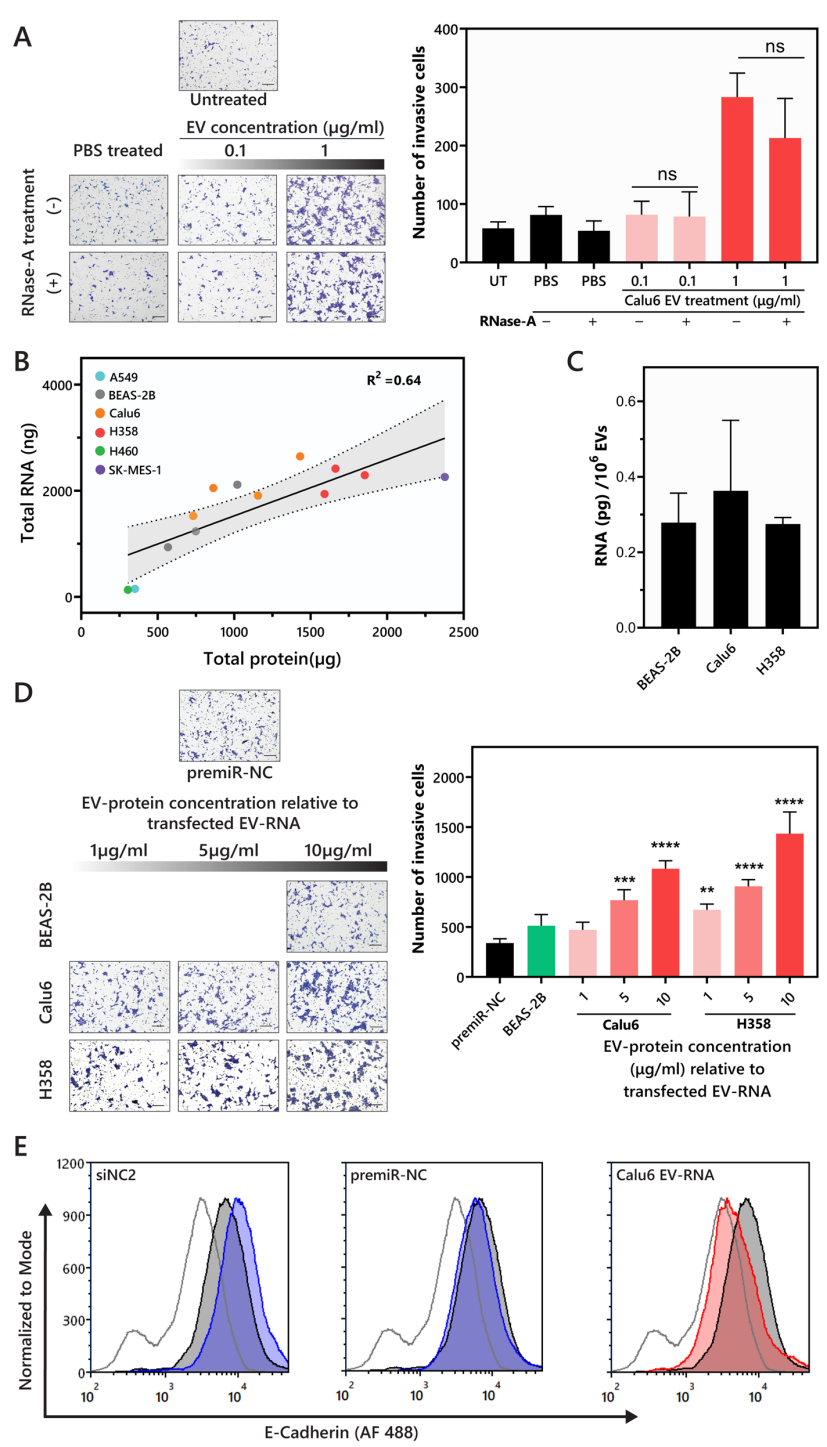
EV-RNA contributes to NSCLC EV-mediated phenotypes. (**A**) Representative images showing the invasive effect of RNase A treated Calu6-EVs on BEAS-2B cells (Scale bar, 200 μm). The number of invasive cells was compared between treatments using unpaired t-test with Welch’s correction. There was no significant difference between BEAS-2Bs incubated with and without RNase A treated Calu6 EVs. Untreated sample is represented as UT (**B**) Correlation between EV protein (µg) and EV-RNA (ng) indicates a strong linear correlation with an R^2^ = 0.64, the dotted line represents the 95% confidence interval for the linear correlation. (**C**) EV-RNA yield per million EVs is not statistically different between tumorigenic (Calu6 and H358) and non-tumorigenic (BEAS-2B) cells. (**D**) Representative images showing the invasive effect of EV-RNA on BEAS-2B cells (Scale bar, 200 μm). EV-RNA concentrations relative to 1, 5, and 10 µg/mL of EV-protein were calculated (see Table S1). BEAS-2B cells were transfected with the indicated *protein-equivalent EV-RNA concentration* and invasion was quantified 48 h following transfection. EV-RNA from NSCLC EVs (Calu6 and H358) significantly increase the number of invasive cells in comparison to premiR-NC. p-values were determined using one-way ANOVA followed by Dunnett’s multiple comparison tests (n = 3, **p < 0.01, ***p < 0.0005 and ****p < 0.0001). (**E**) Representative histograms demonstrate the levels of surface E-cadherin on BEAS-2B cells following transfection with negative control RNA (siNC2 or premiR-NC) or Calu6 EV-RNA relative to 300 µg/mL of EV-protein (n = 2).

The original Article has been corrected.

