# Supplementary material for: Extracellular vesicles released by non-small cell lung cancer cells drive invasion and permeability in non-tumorigenic lung epithelial cells
Source: Sci Rep. 2022 Jan 19;12:972. doi: 10.1038/s41598-022-04940-6 (PMC8770483; doi:10.1038/s41598-022-04940-6)
Supplement: Supplementary file 1 — Supplementary Information. [file 41598_2022_4940_MOESM1_ESM.pdf]

Supplemental Information

Extracellular vesicles released by non-small cell lung cancer cells drive invasion and permeability in non-tumorigenic lung epithelial cells

Humna Hasan[1,†], Ikjot Singh Sohal[1,2,†], Zulaida Soto-Vargas[1,†], Anjali M. Byappanhalli[1], Sean E. Humphrey[1], Hana Kubo[1], Sarunya Kitdumrongthum[3], Sarah Copeland[1], Feng Tian[1], Arthit Chairoungdua[4], and Andrea L. Kasinski[1,2,*]

[1]*Department of Biological Sciences, Purdue University, West Lafayette, IN 47907, USA*

[2]*Purdue Center for Cancer Research, Purdue University, West Lafayette, IN 47907, USA*

[3]*Toxicology Graduate Program, Faculty of Science, Mahidol University, Bangkok Thailand*

[4]*Department of Physiology, Faculty of Science, Mahidol University, Bangkok Thailand*

†Equally contributing authors

*Corresponding author

Andrea L. Kasinski, Ph.D.

akasinski@purdue.edu

Department of Biological Sciences, Purdue University

Figure S1

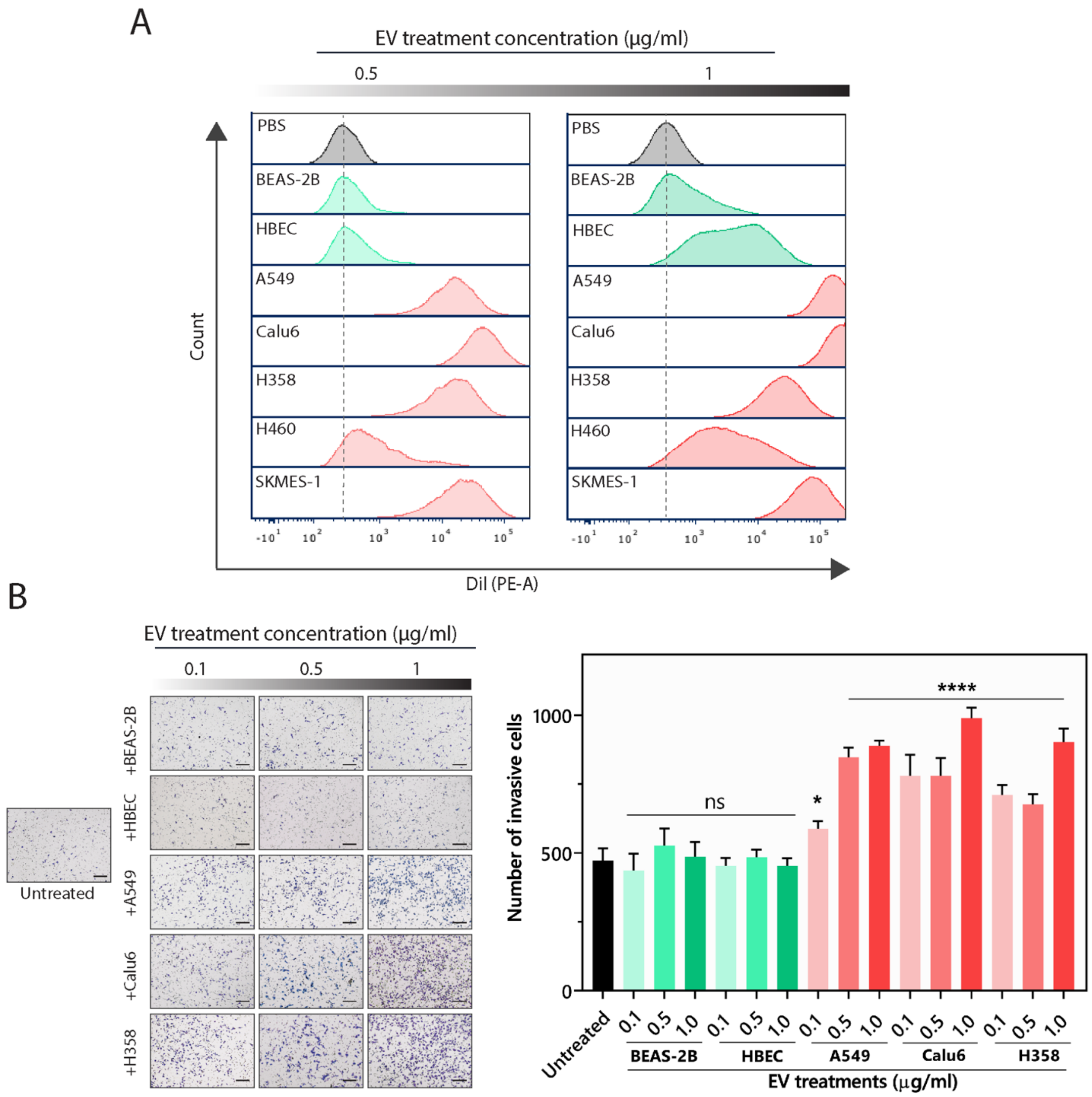

A
EV treatment concentration (µg/ml)
0.5
1
PBS
BEAS-2B
HBEC
A549
Calu6
H358
H460
SKMES-1
Count
Dil (PE-A)
B
EV treatment concentration (µg/ml)
0.1
0.5
1
+BEAS-2B
+HBEC
+A549
+Calu6
+H358
Untreated
Number of invasive cells
1000
500
0
ns
*
****
Untreated
0.1
0.5
1.0
BEAS-2B
HBEC
A549
Calu6
H358
EV treatments (µg/ml)

Figure S1, related to Figure 2.

Induction of invasive phenotype after uptake of NSCLC-derived EVs in human bronchial epithelial cells (HBEC)

(A) Representative histograms of flow cytometry analysis of HBEC cells after treatment with respective DiI-stained EVs for 48 hours (n=1).

(B) Representative images of invasive HBEC cells treated with 0.1μg/ml, 0.5μg/ml or 1μg/ml of tumorigenic (A549, Calu6 and H358) and non-tumorigenic (HBEC and BEAS-2B) cell-derived EVs for 24 hours. NSCLC EVs significantly drive invasion in HBECs compared to untreated control. p-values were determined using one-way ANOVA (n=3, *$p<0.05$, **$p<0.01$ and ****$p<0.0001$). Scale bar, 200 μm.

Figure S2

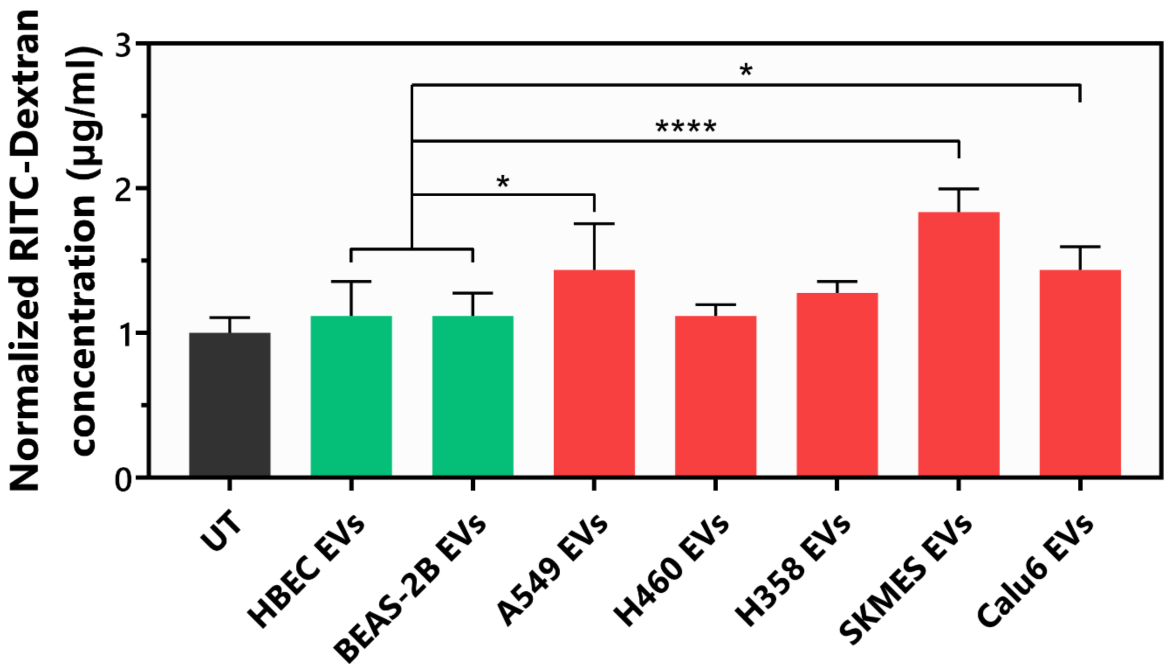

Figure S2, related to Figure 3.

RITC-Dextran permeability across the BEAS-2B epithelial barrier.

The bar graph represents the fold-change in the amount of RITC-Dextran in the basal chamber after 1 hr of the 48 hrs EV treatment (n=3). The UT is normalized to 1 µg/mL. *p<0.05, **p<0.01, ***p<0.001 and ****p<0.00001

Figure S3

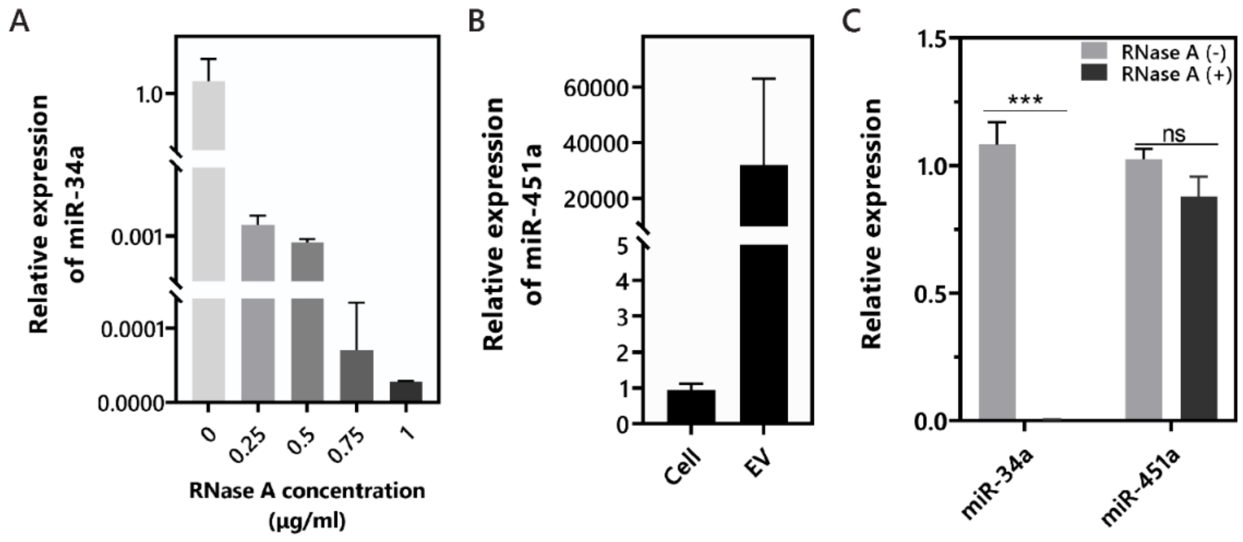

Figure S3, related to Figure 5.

RNase treatments of EVs do not affect EV-RNA and deplete RNA subsets outside EVs

(A) Testing effectiveness of varying concentrations of RNase-A in depleting spiked-in (to represent EV-free RNA) miR-34a (16nM). Relative expression of miR-34a was assessed through qRT-PCR. We chose 0.5 µg/ml concentration of RNase-A for further experiments.

(B) qRT-PCR expression analysis reveals abundance of miR-451 in RNA derived from H358 EVs. miR-451a was used in later experiments to represent EV- RNA.

(C) Treatment of EVs with RNase-A (0.5 µg/ml) followed by RNase inhibitor (RI) (7.5U/ml) causes complete depletion of miR-34a (EV-free RNA) while the levels of miR-451a (EV-RNA) remained unaffected.

Table S1, related to Figure 5D

Concentrations of EV-RNA corresponding to 1, 5 and 10 μg/ml of EV-protein for indicated cell lines. The cells were transfected with a total of 42ng of RNA, therefore, the final RNA concentration for each transfection was supplemented with premiR-NC to keep concentration of RNA consistent in each transfection reaction.

Cell line	EV Protein (μg/ml)	EV-RNA concentration (ng/ml)	premiR-NC (ng/ml)
BEAS-2Bs	10	22	20
Calu6	1	2.5	39.5
	5	12.5	29.5
	10	25	17
H358	1	1.8	40.2
	5	9	33
	10	18	24

Western blots

Figure S4. Biophysical characterization of extracellular vesicles (EVs).

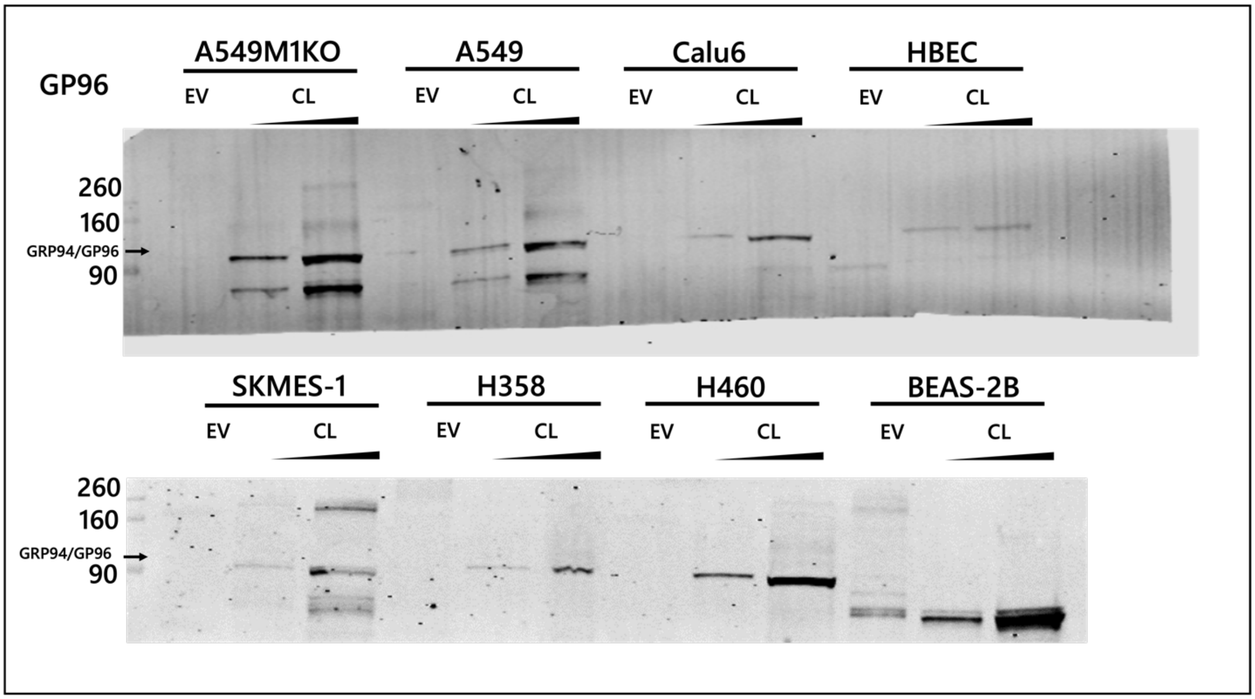

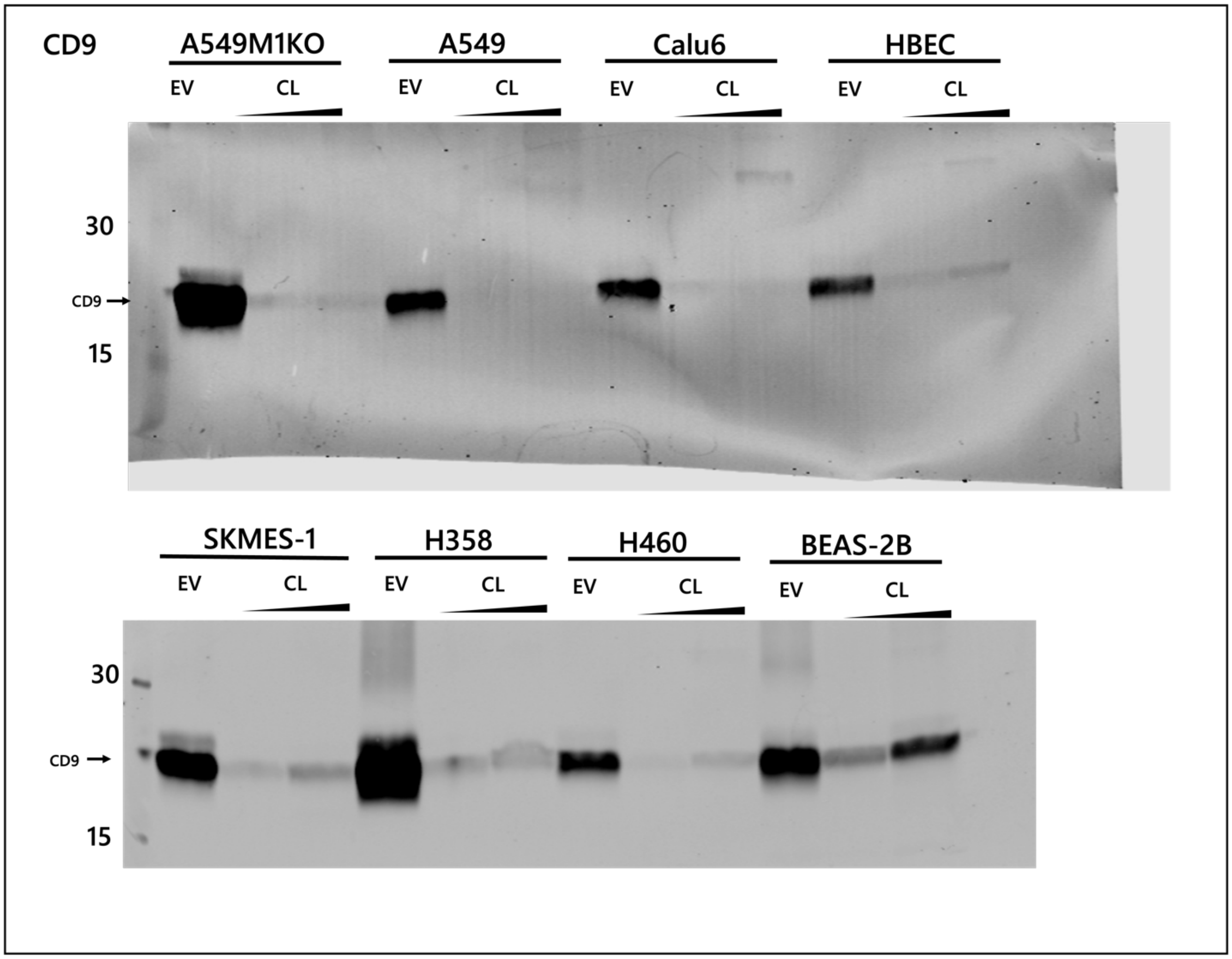
CD9
A549M1KO
A549
Calu6
HBEC
EV
CL
30
CD9
15
SKMES-1
H358
H460
BEAS-2B

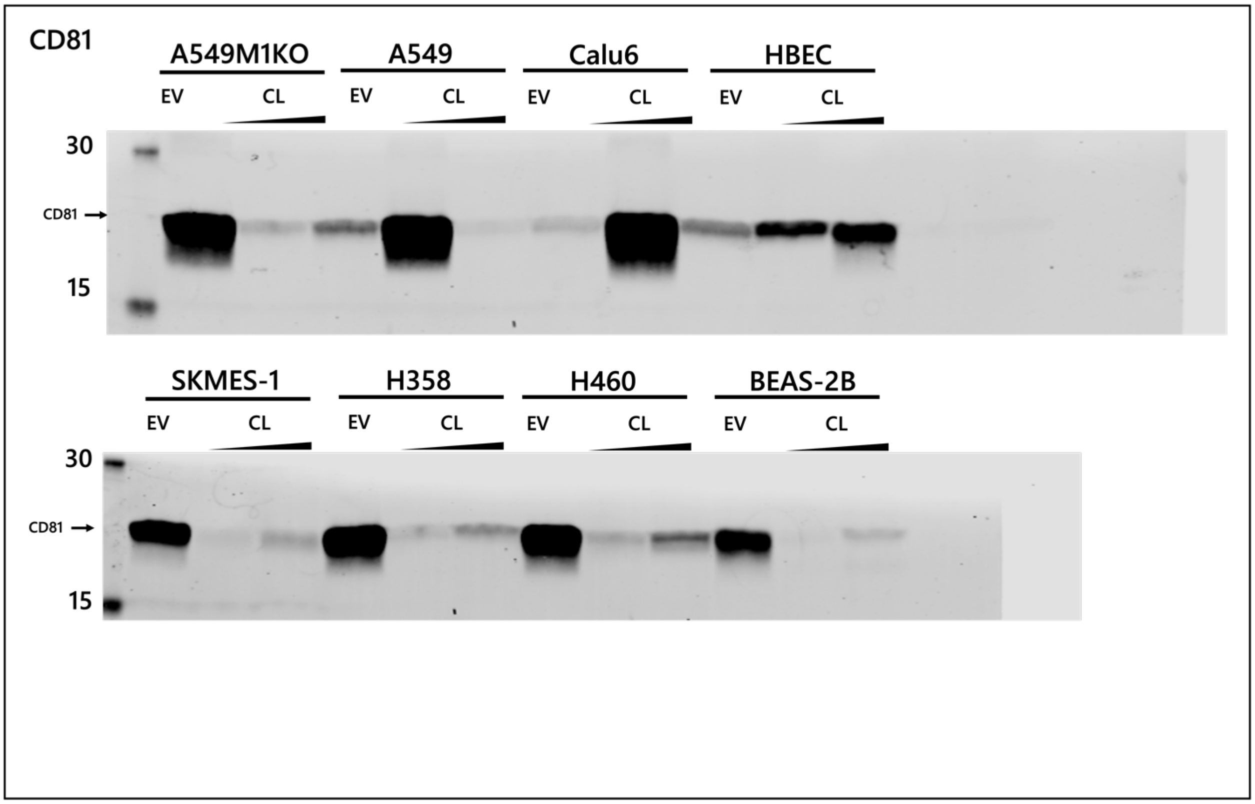
CD81
A549M1KO
A549
Calu6
HBEC
EV
CL
30
CD81
15
SKMES-1
H358
H460
BEAS-2B

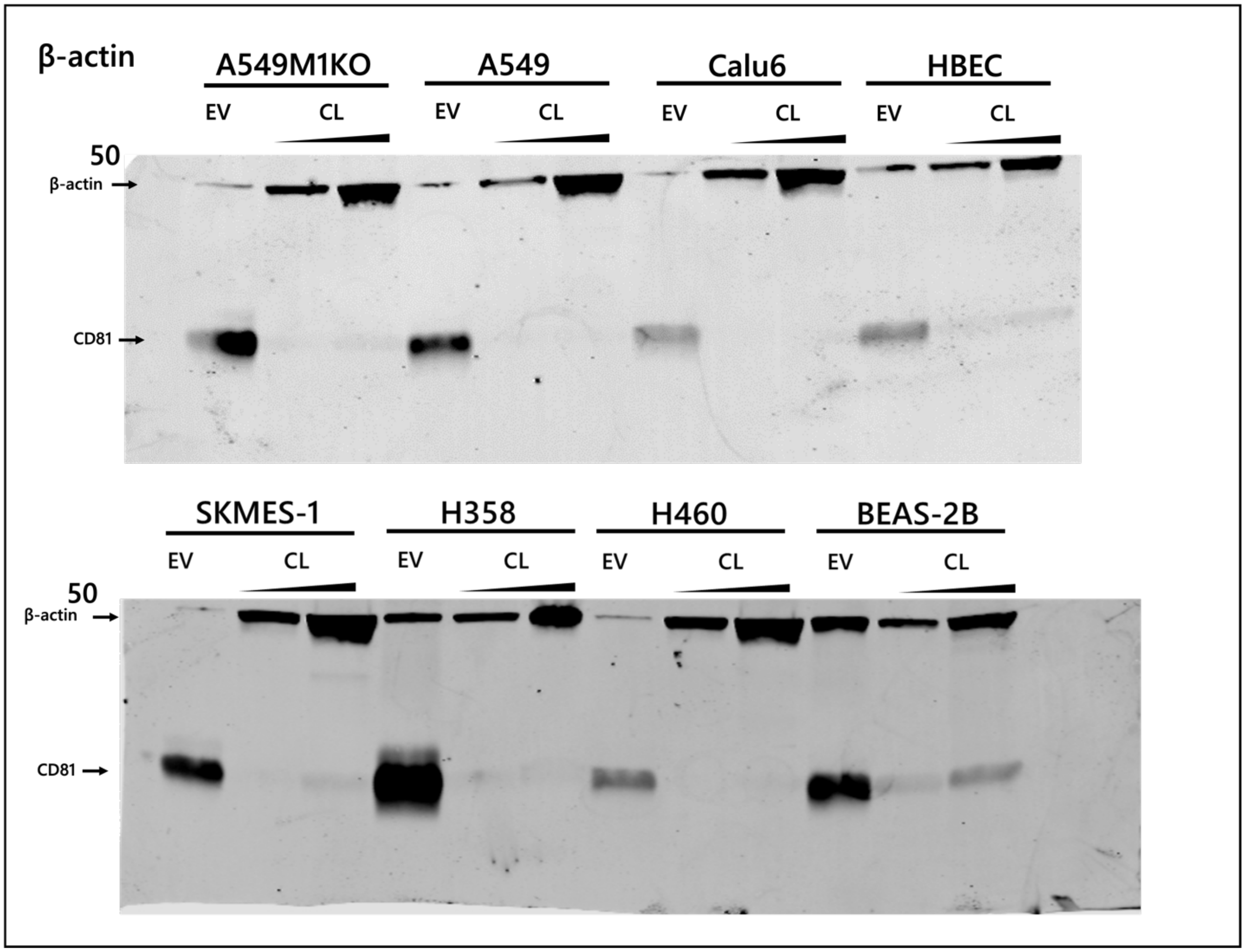
β-actin
A549M1KO
A549
Calu6
HBEC
EV
CL
50
β-actin
CD81
SKMES-1
H358
H460
BEAS-2B

Figure S5. Varied potential of NSCLC EVs to modulate the expression of junctional complex proteins E-cadherin and ZO-1.

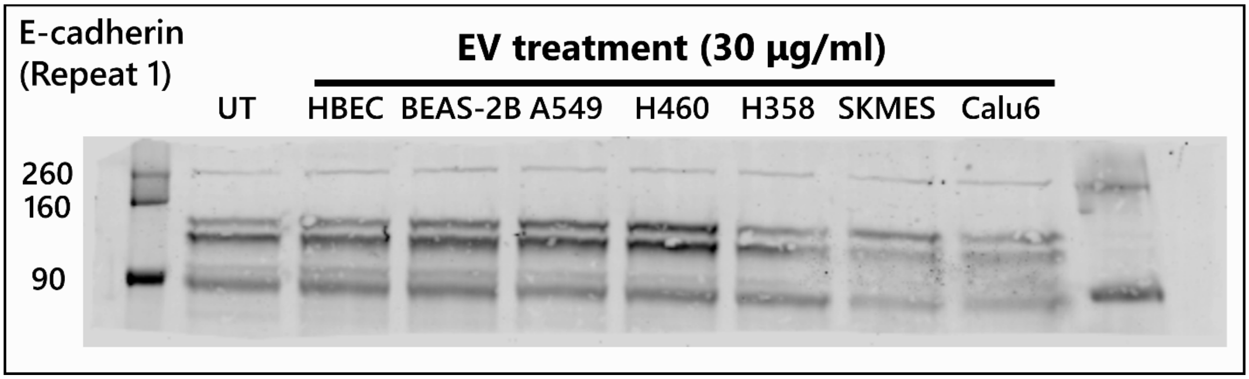

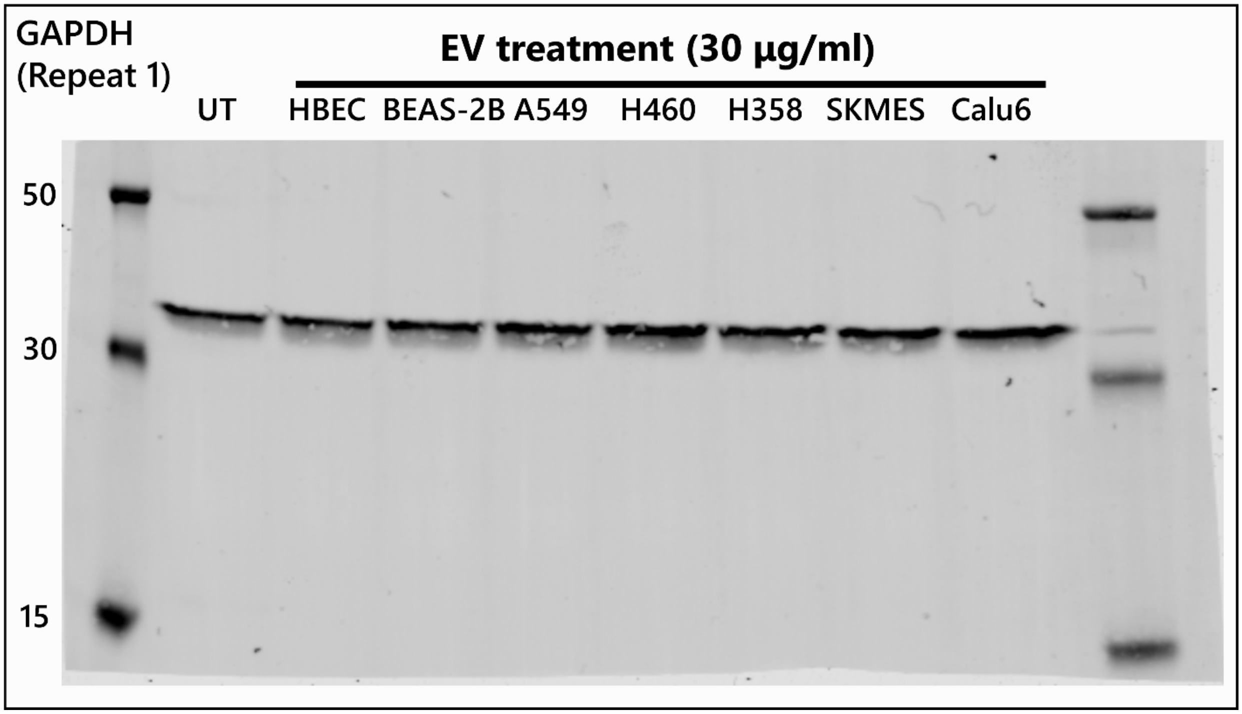

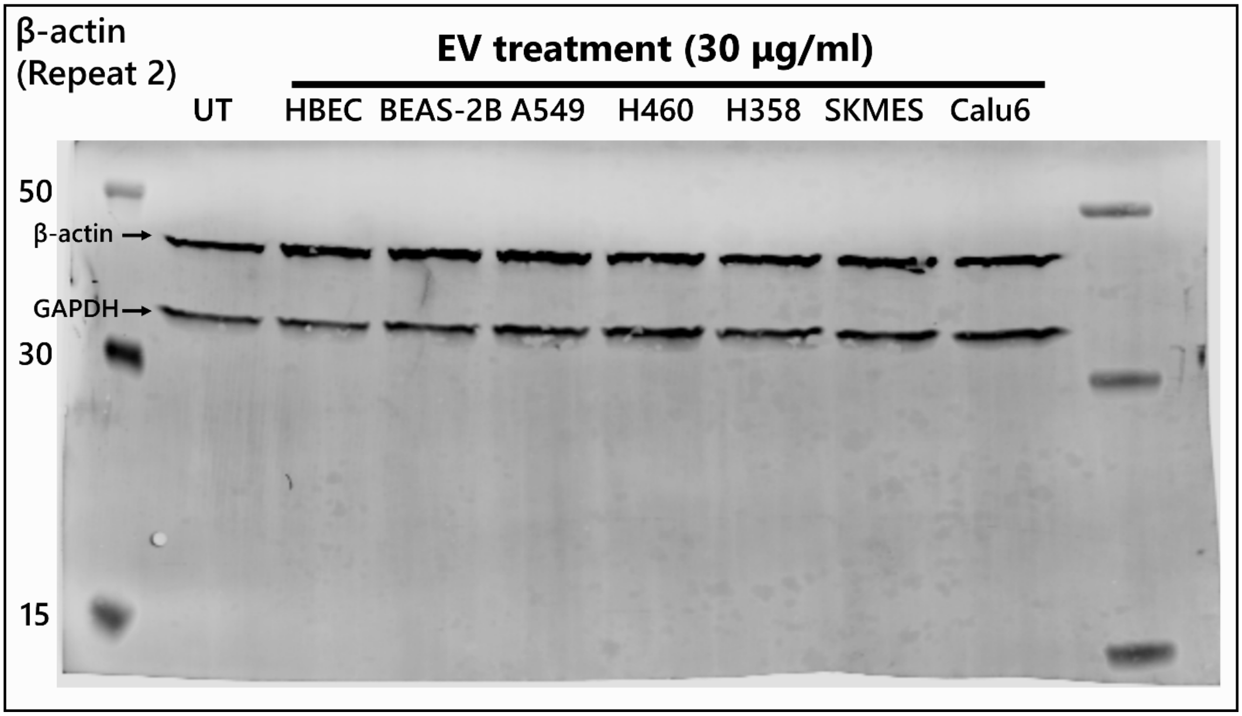
β-actin
(Repeat 2)
EV treatment (30 µg/ml)
UT
HBEC
BEAS-2B
A549
H460
H358
SKMES
Calu6
50
β-actin
GAPDH
30
15

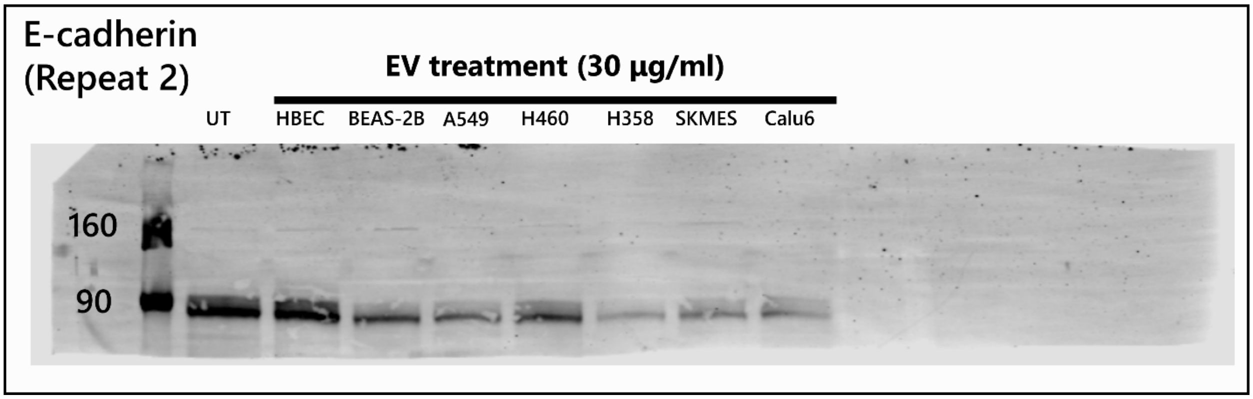
E-cadherin
(Repeat 2)
EV treatment (30 µg/ml)
UT
HBEC
BEAS-2B
A549
H460
H358
SKMES
Calu6
160
90

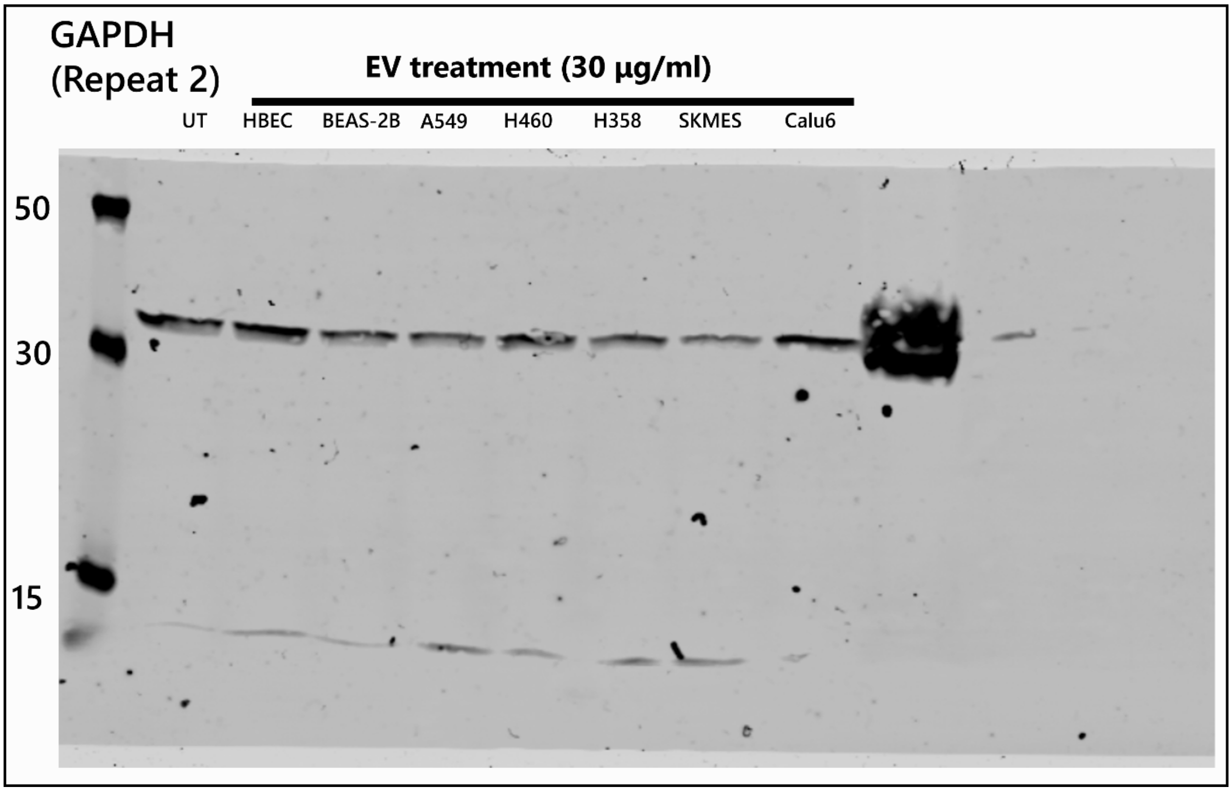
GAPDH
(Repeat 2)
EV treatment (30 µg/ml)
UT
HBEC
BEAS-2B
A549
H460
H358
SKMES
Calu6
50
30
15
